# HNRNPD regulates the biogenesis of circRNAs and the ratio of mRNAs to circRNAs for a set of genes

**DOI:** 10.1080/15476286.2024.2386500

**Published:** 2024-08-24

**Authors:** Shuhui Chang, Yucong Wang, Xiaolin Wang, Hanyuan Liu, Tao Zhang, Yangge Zheng, Xueren Wang, Ge Shan, Liang Chen

**Affiliations:** a Department of Laboratory Medicine, The First Affiliated Hospital of USTC, The RNA Institute, School of Basic Medical Sciences, Division of Life Science and Medicine, University of Science and Technology of China (USTC), Hefei, Anhui, China; b Department of Obstetrics and Gynecology, The First Affiliated Hospital of USTC, Division of Life Sciences and Medicine, University of Science and Technology of China (USTC), Hefei, China; c Department of Urology, the Second Affiliated Hospital of Anhui Medical University, Hefei, China; d Courant Institute of Mathematical Sciences, New York University, New York, USA; e Department of Anesthesiology, Shanxi Bethune Hospital, Taiyuan, China; f Department of Anesthesiology, Tongji Hospital, Tongji Medical College, Huazhong University of Science and Technology, Wuhan, China; g Department of Cardiology, The First Affiliated Hospital of USTC, The RNA Institute, Division of Life Sciences and Medicine, University of Science and Technology of China (USTC), Hefei, Anhui, China

**Keywords:** HNRNPD, circRNA, biogenesis, CDK1, ccRCC

## Abstract

Exonic circular RNAs (ecircRNAs) in animal cells are generated by backsplicing, and the biogenesis of ecircRNAs is regulated by an array of RNA binding proteins (RBPs). HNRNPD is a heterogeneous nuclear ribonucleoprotein family member with both cytoplasmic and nuclear roles, and whether HNRNPD regulates the biogenesis of circRNAs remains unknown. In this study, we examine the role of HNRNPD in the biogenesis of ecircRNAs. The levels of ecircRNAs are primarily increased upon depletion of HNRNPD. HNRNPD preferentially binds to motifs enriched with A and U nucleotides, and the flanking introns of ecircRNAs tend to have more numbers and higher intensity of HNRNPD binding sites. The levels of mRNAs are generally not significantly altered in HNRNPD knockout cells. For a small set of genes, the circRNA:mRNA ratio is substantially affected, and the mRNA levels of some of these genes demonstrate a significant decrease in HNRNPD knockout cells. CDK1 is identified as a key gene modulated by HNRNPD in the context of circRNA biogenesis. HNRNPD suppresses the biogenesis of circCDK1 and favours the generation of CDK1 mRNA, and the CDK1 protein is a critical regulator of the cell cycle and apoptosis. HNRNPD can participate in cellular physiology, including the cell cycle and apoptosis, and plays roles in clear cell renal cell carcinoma (ccRCC) by modulating circRNA biogenesis and the mRNA levels of key genes, such as CDK1.

## Introduction

Circular RNAs (circRNAs) are covalently closed single-stranded RNAs [1,2]. There are multiple classes of circRNAs in eukaryotes, and exonic circRNAs (ecircRNAs) are the major circRNA class in animal cells [3,4]. EcircRNAs are generated from thousands of genes through backsplicing, a form of alternative splicing that links the downstream splice donor and the upstream splice acceptor to generate a circRNA [1,2,4]. For coding genes, the abundance of ecircRNAs is generally lower than that of the corresponding mRNAs, which are generated by conventional linear splicing [5]; the levels of most ecircRNAs are less than 10% of the total transcripts from the same genes [6]. EcircRNAs also exhibit cell-/tissue- specificity [7,8]. Accumulating lines of evidence indicate that backsplicing is generally inefficient or inhibited in splicing, and the biogenesis of ecircRNAs is subjected to precise regulation to manage the ratio of mRNAs to ecircRNAs [5,9–13].

An array of factors have been identified to regulate ecircRNA biogenesis [1,2,4,14]. RNA binding proteins (RBPs), such as Quaking (QKI), Mbl, FUS, RBM20, and HNRNPL, bind to specific motifs in the flanking introns of circularizing exons, and this binding facilitates the approach of the downstream 5’ splice site and the upstream 3’ splice site [10]. Complementary repeat elements, such as Alu elements in human cells and B2 elements in rodent cells, in the flanking introns have been shown to facilitate circRNA biogenesis [15–17]. On the other hand, several inhibitory mechanisms have also been revealed [17–19]. Adenosine deaminase 1 acting on RNA (ADAR1) edits A-to-I in intronic Alu elements, thus leading to the suppression of circRNA biogenesis [17,19]. DHX9 (DEAH-box helicase 9), an RNA helicase, unwinds complementary Alu pairs formed by flanking introns to inhibit circRNA biogenesis [18].

Dysregulation of these RBPs often leads to severe pathological phenotypes [4,14]. For instance, TTN (Titin) produces heart-specific circRNAs whose biogenesis depends on the splicing factor RMB20 [20]. These circRNAs are derived mainly from the coding sequences corresponding to the I-band region of TTN. In RBM20 mutation carriers, alternative splicing of this region is aberrant, and distortion of the formation or function of these circRNAs in RBM20 mutation carriers may contribute to cardiac pathology [21]. Genome-wide CRISPR-Cas9 knockout screening in prostate cancer cells identifies HNRNPL, which directly regulates alternative splicing and circular RNA formation [10]. HNRNPL binding to flanking introns significantly enhances circRNA formation, and HNRNPL knockdown reduces circRNA formation. Both HNRNPL-regulated alternatively spliced genes and HNRNPL-regulated circRNA-generating genes are found to be significantly linked to gene overexpression signatures of prostate cancer in several independent cohorts [10]. QKI enhances circRNA production by binding to recognition elements within flanking introns of circRNA-forming exons, and some QKI-regulated circRNAs are involved in epithelial – mesenchymal transition (EMT) in human cells [22]. Therefore, QKI can regulate cellular properties such as migration and invasion to modulate cancer metastasis [22].

Heterogeneous nuclear ribonucleoprotein D (HNRNPD), also known as AU-rich element (ARE) RNA-binding factor 1 (AUF1), is an RBP with multiple cytoplasmic and nuclear functions in animal cells [23,24]. HNRNPD recognizes U-/GU-rich sequences in mRNAs and long noncoding RNAs (lncRNAs) to decrease the steady-state levels of some RNAs [24]. HNRNPD can also promote polyribosome loading onto some mRNAs and their consequent translation, and the steady-state levels of several mRNAs encoding DNA maintenance proteins are unexpectedly enhanced by HNRNPD [24]. In the nucleus, HNRNPD interacts with the nuclear lncRNA NEAT1 and modulates the levels of NEAT1 and NEAT1-regulated transcripts [24]. HNRNPD forms a complex with the YBX1 protein and the lncRNA TCLlnc1 on the promoters of the TGFB2 and TGFBR1 genes to activate their transcription [25]. HNRNPD interplays with G-quadruplex DNA structures in the *Tert* promoter to promote mTERT expression [26]. In several cases, HNRNPD is known to regulate alternative splicing [27,28]. HNRNPD modulates the alternative splicing of cassette exons in its own 3’ UTR [27]. HNRNPD also acts as a splicing inhibitor of the HPV16 E1/E2- and E6/E7-mRNAs, therefore promoting intron retention (IR) in the E1- and E6-mRNAs by interacting with the components of the splicing machinery [28]. The expression of the HPV16 E7 oncoprotein is enhanced upon knockdown of HNRNPD in HPV16-driven cervical cancer cells, indicating a crucial role of HNRNPD in the development of HPV-associated cancers [28]. In summary, HNRNPD possesses multiple cytoplasmic and nuclear functions in animal cells, and whether and how HNRNPD participates in the backsplicing of circRNA biogenesis have not been examined.

In this study, we explored the roles of HNRNPD in regulating the biogenesis of ecircRNAs. We provided lines of evidence showing that HNRNPD primarily inhibited circRNA biogenesis by binding to intronic sites. Through its regulatory effects on circRNA biogenesis, HNRNPD modulated cellular physiology and participated in renal carcinogenesis.

## Results

### Loss of HNRNPD increases the steady-state levels of EcircRNAs

**Figure 1.**
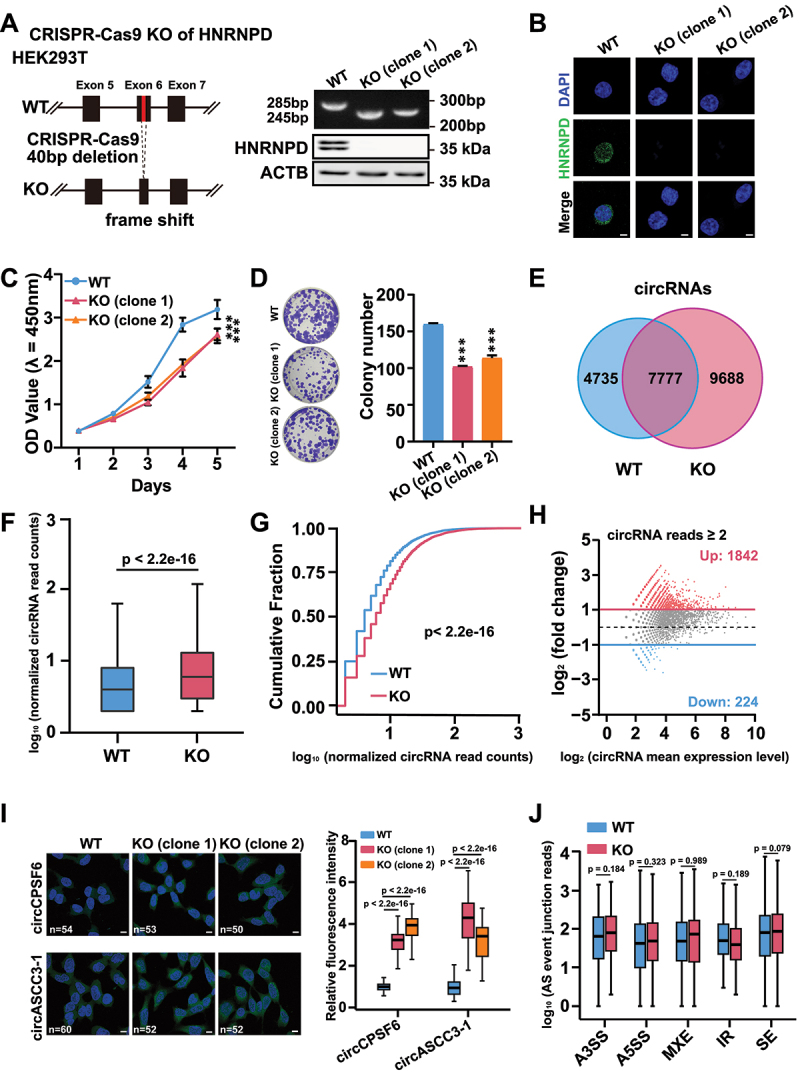
Steady-state level of circRNAs increases upon HNRNPD depletion. (A) Scheme for generation of HNRNPD knockout (KO) HEK293T cells by CRISPR-Cas9 system. Forty nucleotides in exon 6 of HNRNPD were deleted. HNRNPD KO was validated with genomic PCR and western blot (right). β-actin (ACTB) was used as endogenous control. (B) Immunofluorescence (IF) staining of HNRNPD in WT and two KO clone cells. Representative images were shown (DAPI, blue; HNRNPD, green). Scale bar, 20 μm. (C and D) Cell growth was detected by CCK8 and colony formation assay in WT and two KO clone cells. For C, *n* = 5; for D, *n* = 3. (E) Venn diagram showed the overlap of steady level of circRNAs from WT and KO cells. (F and G) The boxplots (F) and the cumulative fraction curves (G) of circRNA levels from WT and KO cells. *n* = 7777. (H) MA plot of the differentially-expressed circRNAs upon HNRNPD KO. circRNAs with at least 2 BSJ reads were used for the analysis. Red plots indicate upregulated circRNAs in HEK293T KO cells. Blue plots indicate downregulated circRNAs in HEK293T KO cells. BSJ, backsplicing junction. (I) Fluorescence *in situ* hybridization (FISH) of circRNAs (circCPSF6 and circASCC3-1) in WT and two KO cells. Representative images were shown (DAPI, blue; circRNA, green). Scale bar, 10 μm. Boxplot showed the relative fluorescence intensity of circRNAs (circCPSF6 and circASCC3-1) in WT and two KO cells. (J) Replicate multivariate analysis of transcript splicing (rMATS) was conducted to analysis the alternative splicing events of the poly(a) enriched transcripts corresponding to differentially-expressed circRNAs in WT and KO cells. SE, skipped exon; A5SS, alternative 5’ splice site; A3SS, alternative 3’ splice site; MXE, mutually exclusive exons; IR, intron retention. The as events with a false discovery rate (FDR) < 0.05 were selected for further analysis. For C, *p* values from the two-way ANOVA test. For D, F and I, *p* values from two-tailed student’s *t* test. For G, *p* value from the Kolmogorov-Smirnov test. For J, *p* values from the likelihood-ratio test. Data are shown as means ± SD from at least three independent experiments. ***p < 0.001.

We first used the CRISPR-Cas9 system to generate HNRNPD knockout (KO) HEK293T cells, a human embryonic kidney cell line (Figure 1(A)). Two HNRNPD knockout clones (clone 1 and clone 2) were generated. In both clones, a fragment of 40 nucleotides was deleted; the deletion was confirmed by genomic PCR, and the lack of HNRNPD protein expression was verified by western blot analysis (Figure 1(A)). HNRNPD has four protein isoforms produced by alternative splicing: p37, p40, p42, and p45 [23,28]. The two major bands (one band as p40 & p42, and the other band as p45) observed in the *wildtype* (WT) cells were not observed for either two KO clone cells (Figure 1(A)). Immunofluorescence (IF) staining also confirmed HNRNPD depletion in the two KO clones (Figure 1(B)). An evident phenotype of the KO cells was a reduced rate of cell growth (Figure 1(C,D)), and mechanistic insights into this phenotype were later provided.

High-throughput RNA sequencing (RNA-seq) analysis of the WT and HNRNPD KO cells was then performed. In the RNA-seq data 12,512 ecircRNAs from WT cells and 17,465 from KO cells were identified, among which the 7,777 circRNAs identified in both cell lines were subjected to further analyses (Figure 1(E)). We focused on ecircRNAs, and all content in this study related to circRNAs actually relates specifically to ecircRNAs. For the next-generation RNA-seq analysis, the sequencing depth and detection efficiency of circRNAs were lower than those of linear RNAs [29,30], and we chose these 7,777 overlapping circRNAs as high-confidence circRNAs for further analyses (Supplementary Table S1). Compared with those in WT cells, the levels of these circRNAs in KO cells were significantly increased (Figure 1(F,G)), indicating a suppressing role of HNRNPD in circRNA levels.

Among the 2,066 differentially expressed circRNAs in the KO cells, 1,842 circRNAs (~89.2%) were significantly upregulated (Fold change ≥ 2), and 224 circRNAs (~10.8%) were significantly downregulated (Fold change ≤ 0.5) (Figure 1(H) and Supplementary Table S1). Nine circRNAs with differential expression were selected for experimental validation via RT-qPCR using divergent primers (Supplementary Table S2 and Supplementary Fig. S1 a – c). Their corresponding mRNAs were also examined, which were either not affected or were changed to a lesser degree than the changes observed for the circRNAs upon HNRNPD depletion (Supplementary Fig. S1 a – c). Nine circRNAs with significantly upregulated levels and two circRNAs (circDnmt1 and circPCNX1) without significant changes in the KO cells according to RNA-seq analysis were then selected for examination; we found that HNRNPD overexpression in the two KO clones led to decreased levels of the nine circRNAs but not the two negative control circRNAs, while the levels of their corresponding mRNAs were mostly not affected or slightly changed (Supplementary Fig. S1d). Two examples in these nine circRNAs, namely, circCPSF6 and circASCC3–1, were also examined by fluorescence *in situ* hybridization (FISH), and higher circRNA FISH signals were displayed in the two KO clones than in the WT cells (Figure 1(I)). Furthermore, alternative splicing of linear transcripts upon HNRNPD depletion was examined by sequencing of poly(A)-enriched transcripts (Supplementary Fig. S1e). Out of the five canonical alternative splicing events, skipped exon (SE), alternative 5’ splice site (A5SS), alternative 3’ splice site (A3SS), mutually exclusive exons (MXE), and IR events, only IR events were significantly affected, with reduced levels in KO cells (Supplementary Fig. S1e). Additionally, alternative splicing events of linear transcripts corresponding to the 2,066 differentially expressed circRNAs in KO cells were not significantly altered (Figure 1(J)), indicating that the impact of HNRNPD knockout on circRNAs was through backsplicing, but was not linked to canonical alternative splicing. Taken together, these data demonstrated that HNRNPD was a suppressor that inhibited steady levels of more than a thousand of ecircRNAs.

### Depletion of HNRNPD promotes EcircRNA biogenesis

**Figure 2.**
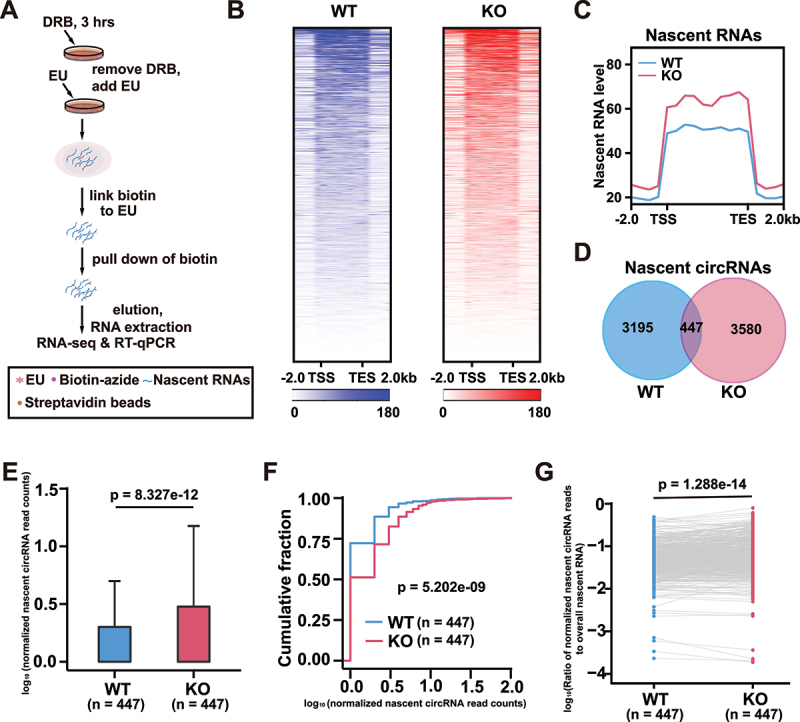
Depletion of HNRNPD enhances circRNA biogenesis. (A) Schematic illustration of EU-labeled nascent RNAs purification and measurement. (B) Heatmap represented the normalized level of nascent RNAs in WT and KO cells. The color bar showed the level of nascent RNA. (C) The level of nascent RNAs in WT and KO cells. TSS, transcription start site; TES, transcription end site. (D) Venn diagram showed the overlap of nascent level of circRNAs from WT and KO cells. (E and F) the boxplots (E) and the cumulative fraction curves (F) of nascent level of circRNA from WT and KO cells. (G) The comparison of ratios of nascent circRNAs to the overall nascent RNA reads from the same genes. For E and G, *p* values from two-tailed student’s *t* test. For F, *p* value from the Kolmogorov-Smirnov test.

To explore the effects of HNRNPD depletion on nascent RNAs, we captured newly transcribed RNAs labelled with 5-ethyluridine (EU) in WT and KO cells with streptavidin-conjugated beads [31] (Figure 2(A)). 557 and 626 genes in the WT and KO cells, respectively, gave rise to the identified nascent RNAs (Figure 2(B)). Nascent RNAs were significantly more abundant in KO cells than in WT cells (Figure 2(C)). For circRNAs, 3642 and 4027 nascent circRNAs were identified in WT and KO cells, respectively (Figure 2(D)). The 447 overlapping nascent circRNAs were chosen for further analyses (Supplementary Table S3). Nascent circRNA levels were increased (Figure 2(E,F)). Furthermore, when the ratios of the nascent circRNAs to the overall nascent RNA reads from the same genes were compared, significantly higher proportions of circRNAs were found to be generated in KO cells from the genes that exhibited both linear splicing and backsplicing (Figure 2(G)). Validation of several examples of nascent circRNAs and their corresponding nascent mRNAs were consistent with the nascent RNA-seq data (Supplementary Fig. S2a). HNRNPD overexpression in the two KO cell lines led to decreased nascent levels of the 8 circRNAs examined, while the levels of the corresponding mRNAs were not significantly affected (Supplementary Fig. S2b).

HNRNPD is known to regulate mRNA degradation via ARE-mediated decay [23]. We thereby tested whether HNRNPD depletion resulted in circRNA degradation. No significant changes in the levels of several examples of circRNAs were observed between the WT and KO cells when the cells were treated with actinomycin D to inhibit transcription and to evaluate RNA degradation (Supplementary Fig. S2 c – i). Collectively, our results demonstrated that depletion of HNRNPD led to increased levels of ecircRNAs, primarily by enhancing circRNA biogenesis.

### HNRNPD preferentially binds to intronic sites of pre-mRNAs

**Figure 3.**
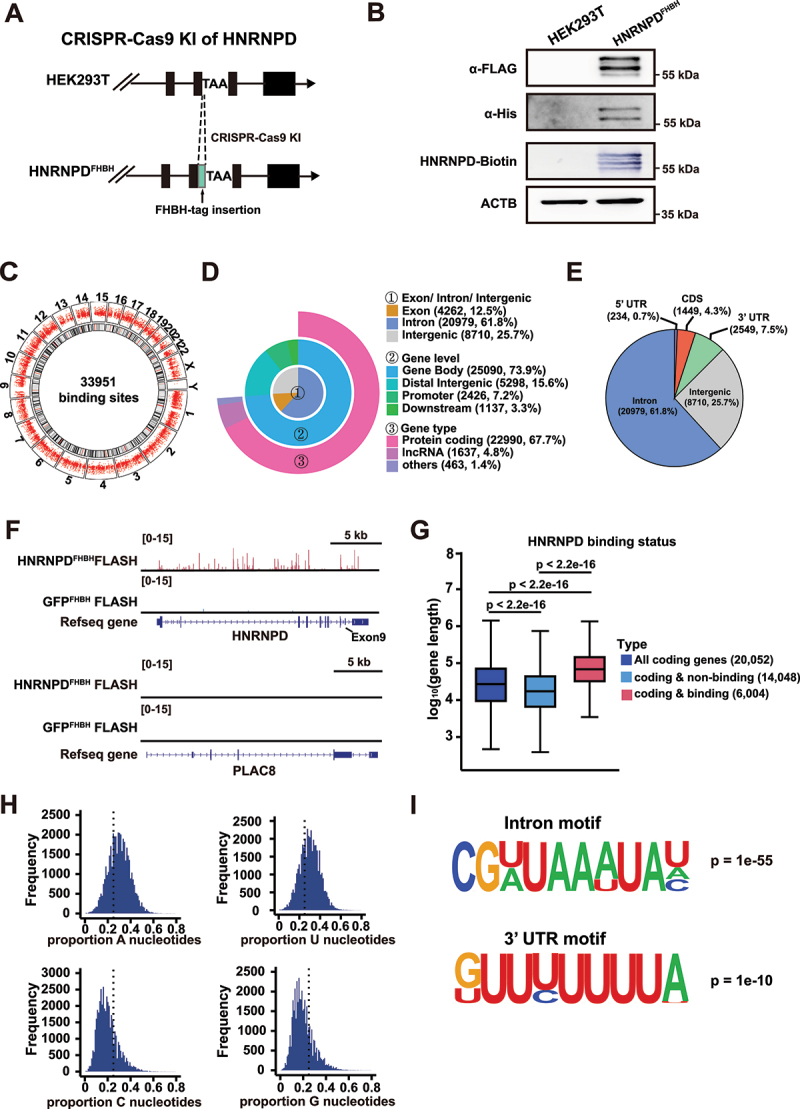
HNRNPD binds to intronic sequences of pre-mRNAs. (A) Scheme for generation of HNRNPD^FHBH^ cell line. The FHBH tag was inserted before the stop codon of HNRNPD genomic DNA by CRISPR-Cas9 knockin. (B) Western blot validation for HNRNPD^FHBH^ protein. HNRNPD has four isoforms: p37, p40, p42, and p45. Detection of four bands or two major bands (one band for p40 & p42, and the other band for p45) was shown. (C) Circos plots exhibited the number and genomic distribution of HNRNPD binding sites. (D) Distribution of HNRNPD binding peaks across different genomic features. The number and the proportion of each part were indicated in the legend. (E) Distribution of HNRNPD binding sites in genomic regions. The number and the proportion of HNRNPD binding sites were indicated. (F) Snapshot of the genomic region with HNRNPD binding. The RefSeq genes was shown in the bottom of the snapshots. Group transfected with GFP^FHBH^ was used as the negative control. Kemmerer et al. [27] identified HNRNPD controlled alternative splicing of its exon 9. (G) Comparison of the length of coding genes with or without HNRNPD binding showed by boxplot. *n* = number of genes detected. (H) Histogram plot of nucleotide frequency within high-confidence hnrnpd-binding peaks (*p* < 0.05, reads > 5). (I) Motif analysis of hnrnpd-binding peaks in the intronic and 3’ UTR region of genes. For G, *p* values were from the *Wilcoxon* test.

To further reveal the features of HNRNPD binding to RNAs, we performed a FLASH assay (Supplementary Fig. S3a), which can be used to identify protein-RNA binding sites in living cells [32,33]. The FLASH assay presents single-nucleotide resolution and specificity without the use of radioactive substances or the isolation of RNA from nitrocellulose filters [32]. A DNA fragment encoding a 110 amino acid (AA) tag was inserted into the 3’ end of the HNRNPD coding sequence using CRISPR-Cas9, which led to the generation of an HNRNPD fusion protein with a C-terminal FHBH tag. The FHBH tag consisted of 3×FLAG, 6×his, the 75 AAs that can be biotinylated, and another 6×his (Figure 3(A)). HNRNPD with the full-length FHBH tag was successfully expressed, and the 75 AAs from the 1.3S biotin-containing subunit of *Propionibacterium shermanii* transcarboxylase were biotinylated as expected *in vivo* by the metabolic biotinylation system in mammalian cells (Figure 3(B)). The GFP^FHBH^ cell line was used as a negative control for the FLASH assay.

By subsequent FLASH assays using His-tag pulldown beads and streptavidin beads with the HNRNPD^FHBH^ cell line 33,951 HNRNPD binding sites were identified at the whole-genome level with high confidence (*p* < 0.05, reads > 5) (Figure 3(C)). Approximately 67.7% of the HNRNPD binding sites were mapped to 6,004 protein-coding genes (Figure 3(D)), and for the protein-coding genes, ~61.8% of the binding sites fell within intronic regions (Figure 3(E)). HNRNPD binding sites mapped to its own gene body were shown as a positive example, as it is known that HNRNPD can regulate the alternative splicing of its own mRNA [27] (Figure 3(F)). There was no HNRNPD binding site mapped to PLAC8, an adjacent gene of HNRNPD, as a reference to demonstrate the specificity of FLASH (Figure 3F). HNRNPD binding sites mapped to several other circRNA generated genes were also demonstrated as examples (Supplementary Fig. S3 b – f). The 9 circRNAs with significantly higher levels and the two circRNAs without significant changes in the HNRNPD KO cells were then examined in KO cells expressing HNRNPD-FHBH (Supplementary Fig. S3g). Expressing HNRNPD-FHBH in the HNRNPD KO cells led to significantly decreased levels of these nine circRNAs, and this effect was similar to that of expressing *wildtype* HNRNPD in the KO cells (Supplementary Fig. S3g and Fig. S1d). We also examined cell growth after expressing HNRNPD-FHBH in the HNRNPD KO cells, and the slower cell growth phenotype of HNRNPD KO cells was rescued upon HNRNPD-FHBH expression (Supplementary Fig. S3h). These results indicated that HNRNPD-FHBH was functional.

We observed that the length of the coding genes with HNRNPD binding sites was significantly longer than that of the coding genes without HNRNPD binding sites and that of the overall coding genes analysed (Figure 3(G)). HNRNPD binding sites exhibited preferences to adenine (A) and uridine (U) nucleotides, consistent with the previously reported HNRNPD binding preference for AREs in the 3’ UTR [23] (Figure 3(H)). We then analysed the HNRNPD binding motifs in the intronic regions and in the 3’ UTR from the FLASH data, and it showed that HNRNPD preferred different sequences in introns and 3’ UTR regions, with a highly U enriched 3’ UTR motif and a U/A enriched intronic motif (Figure 3(I)). The tendency of HNRNPD tends to bind to intronic U/A-enriched sites was previously unknown.

### HNRNPD binding sites have a higher presence in the flanking introns of circRNAs

**Figure 4.**
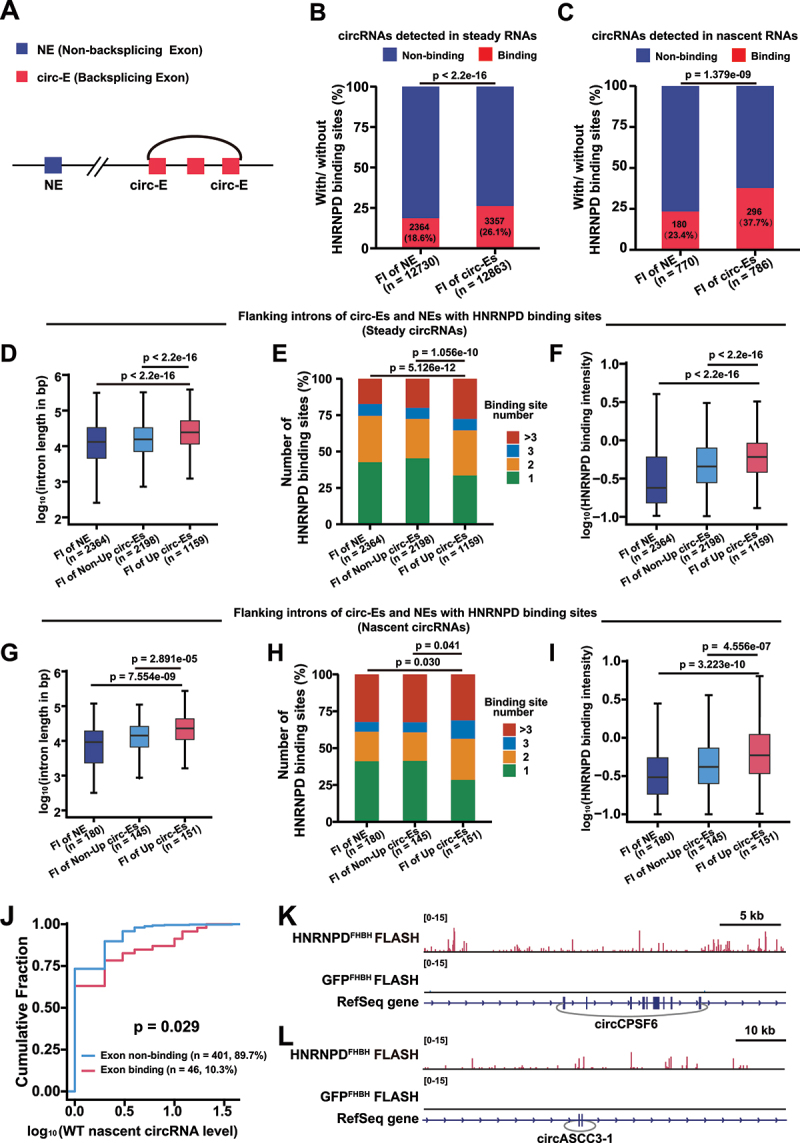
HNRNPD binding sites have more presence in the flanking introns of circRNAs. (A) Scheme for non-backsplicing exon (NE) and backsplicing exon (circ-E). (B) Percentage of flanking introns (FI) with or without HNRNPD binding for NEs and circ-Es. circ-Es were from the corresponding 7,777 steady circRNAs, while NEs were from the same set of circRNA generating genes. (C) Percentage of FI with HNRNPD binding for NEs and circ-Es. circ-Es were from the corresponding 447 nascent circRnas, while NEs were from the same set of circRNA generating genes. (D – F) Comparison of the length (D), number of HNRNPD binding sites (E), HNRNPD binding intensity (F) of FI with HNRNPD binding for NEs and circ-Es. Up circ-Es are from circRNAs with increased steady level (Fold change ≥ 2) in HNRNPD KO HEK293T cells. Non-up circ-Es are from circRNAs with decreased or unaltered steady level (Fold change < 2) in HNRNPD KO HEK293T cells. (G – I) Comparison of the length (G), number of HNRNPD binding sites (H), HNRNPD binding intensity (I) of FI with HNRNPD binding for nascent NEs and circ-Es. Up circ-Es are from circRNAs with increased nascent level (Fold change ≥ 1.4) in HNRNPD KO HEK293T cells. Non-up circ-Es are from circRNAs with decreased or unaltered nascent level (Fold change < 1.4) in HNRNPD KO HEK293T cells. (J) Cumulative fraction curves were shown the level of WT nascent circRNAs with or without exonic HNRNPD binding. (K and L) Snapshot of the genomic region of CPSF6 (K) and ASCC3-1 (L) with HNRNPD binding. The RefSeq gene was shown in the bottom of the snapshots. The circRNA backsplicing junction was connected by grey arc. GFP^FHBH^ group was used as the negative control. For B, C, E and H, *p* values were from chi-square test. For D, F, G and I, *p* values were from two-tailed student’s *t* test. For J, *p* value was from the Kolmogorov-Smirnov test.

Flanking introns of ecircRNAs generated by backsplicing are known to participate in circRNA biogenesis [16,17]. To analyse the binding features of HNRNPD to flanking introns of ecircRNAs, we evaluated the HNRNPD binding sites in both flanking introns of exons that were involved in backsplicing (circ-E), and exons that were not involved in backsplicing (NE) but also from the same set of circRNA generating genes, were analysed for comparison (Detailed information is provided in the Methods section ‘Identification of the HNRNPD Binding Sites in the Flanking Introns of circRNAs’) (Figure 4(A)). For ecircRNAs detected either in the steady-state RNAs or in the nascent RNAs, flanking introns of circ-Es were more likely to possess HNRNPD binding sites than were those of NEs (Figure 4(B,C)). We then analysed the flanking introns of circ-Es and NEs with HNRNPD binding sites and found that the lengths of these flanking introns and the features of the HNRNPD binding sites were different (Figure 4(D–I)). The flanking introns were longest for those circ-Es corresponding to the upregulated circRNAs in the steady-state RNAs of KO cells (Figure 4(D)), and they tended to possess more numbers and higher intensity of HNRNPD binding sites than those of NEs or circ-Es corresponding to the circRNAs not upregulated in the steady-state RNAs of the KO cells (Figure 4(E,F)). These results held true for the flanking introns of those circ-Es corresponding to the upregulated circRNAs in the nascent RNAs of KO cells (Figure 4(G–I)).

Of note, we observed that HNRNPD binding sites were also present in the circularized exons of a small set of ecircRNAs; 46 (out of the 447, ~10.3%) ecircRNAs detected in the nascent RNAs had HNRNPD binding sites in their composing exons (Figure 4(J)). The levels of nascent circRNAs in WT cells with exonic HNRNPD binding were significantly higher than those of circRNAs without exonic HNRNPD binding (Figure 4(J)). These results indicated that HNRNPD binding to exons might have regulatory roles in the expression levels of these circRNAs, although this possibility requires further investigation. CircCPSF6 and circASCC3–1 were two examples of circRNAs with HNRNPD binding signals mapped to the corresponding exons that were composed of the circRNA (Figure 4(K,L)). In pulldown assays, specific antisense oligos targeting circCPSF6 or circASCC3–1 could co-pull down the HNRNPD protein, and in RIP experiments, anti-HNRNPD antibodies also pulled down circCPSF6 and circASCC3–1 (Supplementary Fig. S4 a – f). Interestingly, anti-HNRNPD antibodies also pulled down the CPSF6 and ASCC3 mRNAs (Supplementary Fig. S4f). The binding of HNRNPD on a small set of exons requires further investigation of its functionality.

Taken together, these data indicated that more numbers and a higher intensity of HNRNPD binding sites in the relatively longer flanking introns of ecircRNAs played inhibitory roles in circRNA biogenesis.

### HNRNPD can function by modulating the ratio of CircRNAs to mRNAs for particular genes

**Figure 5.**
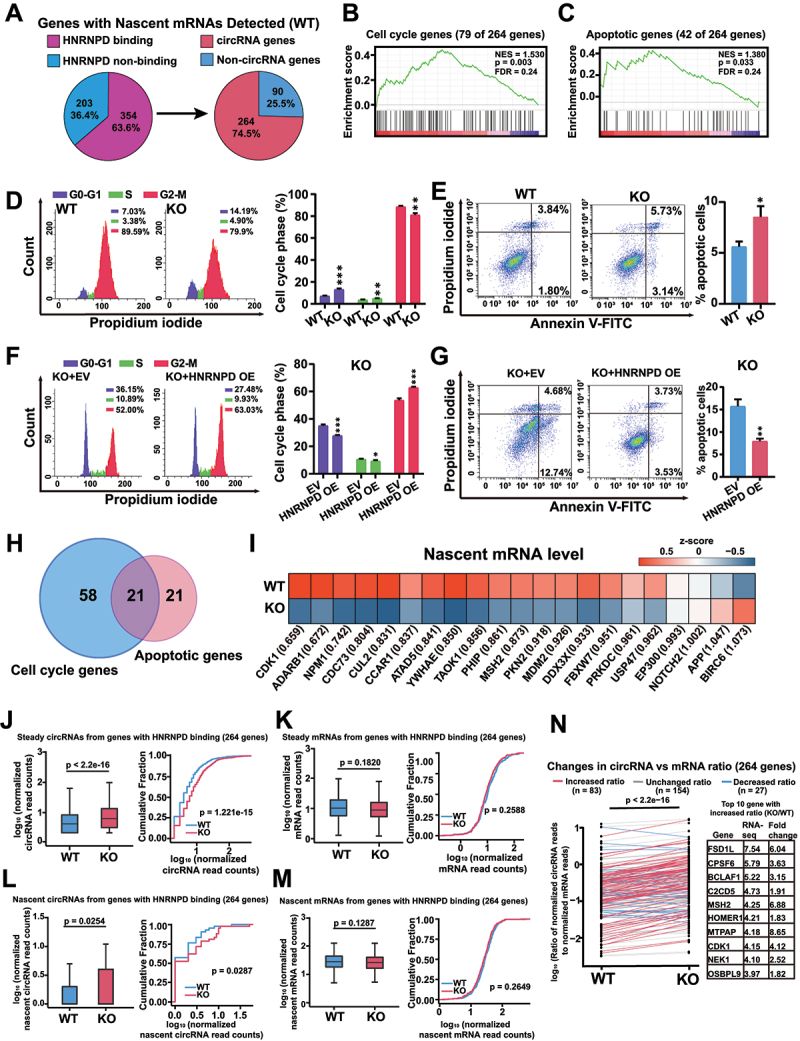
HNRNPD modulates the ratio of circRNAs and mRNAs of specific genes. (A) The genes that had nascent mRNA reads detected in WT cells. The percentage of these genes with HNRNPD binding sites from FLASH analysis (left). The percentage of the genes both possessing HNRNPD binding sites and generating circRNAs (right). (B and C) Gene set enrichment analysis (GSEA) demonstration of the enriched pathways for the 264 genes from figure 5(A). (D and E) Flow cytometry was performed to detect cell cycle and cell apoptosis of WT and KO cells. (F and G) Cell cycle and apoptosis of HNRNPD KO cells after HNRNPD overexpression. EV, empty vector. HNRNPD OE, HNRNPD-FLAG overexpression. (H) Venn diagram showed the overlap of the genes relating to cell cycle and apoptosis pathway according to GSEA. (I) Heatmap of the nascent mRNA level of the overlapped genes. Nascent RNA expression level is normalized to Z-score. Fold change of nascent RNA levels of individual gene in HNRNPD KO HEK293T cells compared to WT cells is included in the brackets. (J and K) Bioinformatics analysis of the steady level of circRNAs and corresponding mRNAs from 264 genes in WT and KO cells. The box plot (left) and the cumulative fraction curves (right) demonstrated the steady level of circRNAs (J) and the corresponding mRNAs (K) in WT and KO cells. (L and M) Bioinformatics analysis of the nascent level of circRNAs and corresponding mRNAs from 264 genes in WT and KO cells. The box plot (left) and the cumulative fraction curves (right) demonstrated the nascent level of circRNAs (L) and the corresponding mRNAs (M) in WT and KO cells. (N) The comparison of ratios of normalized circRNA reads to the normalized mRNA reads from these 264 genes. The ratio of RNA-seq was calculated by KO/WT. The increased ratio >2; the decreased ratio < 1; the unchanged ratio ≥ 1 and ≤ 2. Detailed information is provided in the methods section ‘calculation of the fold change of circRNA: mRNA ratio’. For d – g, *n* = 3. For d – g, and j – m (left panels), *p* values from two-tailed student’s *t* test. For J – M (right panels), *p* values from the Kolmogorov-Smirnov test. Data are shown as means ± SD from three independent experiments. *p < 0.05, **p < 0.01, and ***p < 0.001.

We next focused on the analyses of genes with nascent mRNAs detected in the WT cells, speculating that these genes were transcribed at relatively high levels, and therefore could provide a unique aspect to probe the functional roles of HNRNPD. A total of 557 genes had nascent mRNA reads detected in WT cells, and the FLASH assay identified HNRNPD binding sites mapped to 354 (63.6%) of these genes (Figure 5(A) and Supplementary Fig. S5a). 264 (74.5%) out of these 354 genes generated circRNAs detected in the steady-state RNA-seq analysis (Figures 1(E), 5(A) and Supplementary Fig. S5a). GSEA revealed that these 264 genes were enriched in the cell cycle and apoptotic pathways (Figure 5(B,C)). Cell cycle analysis and apoptosis analysis demonstrated that more cells were at the G0-G1 phase, and more apoptotic cells were present in KO cells (Figure 5(D,E)). Rescue experiments by expressing HNRNPD in KO cells could block the effects on the cell cycle and apoptosis (Figure 5(F,G)). Twenty-one genes overlapped between the genes enriched in the cell cycle pathway and the genes enriched in the apoptotic pathway, and CDK1 (cyclin-dependent kinase 1) exhibited the most significant change in the nascent mRNA level (Figure 5(H,I)).

On the other hand, when examined as a whole, steady-state levels of circRNAs from these 264 genes were increased in the KO cells compared with the WT cells (Figure 5(J)), while no significant difference was observed in the steady-state levels of the corresponding mRNAs (Figure 5(K)). Nascent levels of circRNAs that derived from these 264 genes were also significantly increased in KO cells compared to WT cells, while as a whole the nascent mRNA levels again showed no significant change (Figure 5(L,M)). When the changes of circRNA versus mRNA (circRNA: mRNA ratio) at the steady-state levels from the above 264 genes were examined, 83 genes showed an increased ratio (change in the circRNA:mRNA ratio > 2) in the KO cells, and 27 genes had a decreased ratio (change in the circRNA:mRNA ratio < 1) in the KO cells (Figure 5(N)). The top ten genes exhibiting the highest increase in the circRNA:mRNA ratio upon HNRNPD depletion were examined after HNRNPD overexpression in KO cells, and the corresponding ratios were decreased (Supplementary Fig. S5b). Two genes, CDK1 and MSH2, from the above ten genes were also among the genes involved in apoptosis and the cell cycle in the GSEA (Figure 5(N)). We therefore investigated CDK1, as it is a well-studied and key gene in controlling the cell cycle and apoptosis [34–37]. For both the steady-state and nascent levels, circCDK1 was significantly upregulated, and the levels of CDK1 mRNA and protein were conversely downregulated upon HNRNPD depletion (Supplementary Fig. S5 c – e). In contrast, overexpression of HNRNPD in KO cells led to decreased steady-state and nascent levels of circCDK1 and increased steady-state and nascent levels of CDK1 mRNA (Supplementary Fig. S5 f and g). This regulation of cell cycle- and apoptosis-related genes, including CDK1, by HNRNPD could also explain the phenotypes observed in Figures 1(C,D). The biogenesis of ecircRNA and mRNA compete against each other [9]. Although these analyses indicated that the mRNA levels of most genes were not significantly regulated by HNRNPD in the context of circRNA biogenesis, HNRNPD could still have potent regulatory effects on the expression of key genes such as CDK1 by modulating the ratio of linear splicing to backsplicing.

CDK1 is the major protein kinase that drives cells into mitosis and is associated with apoptosis inhibition [36,37]. Subsequent functional studies demonstrated that knockdown of CDK1 with siRNAs in WT cells led to decreased cell cycle progression and enhanced cell apoptosis (Supplementary Fig. S5 h and i), while overexpression of circCDK1 in WT cells had no effect on cell proliferation or cell apoptosis (Supplementary Fig. S5 j and k). Overexpression of the CDK1 protein in KO cells promoted cell cycle progression and decreased cell apoptosis (Supplementary Fig. S5 l and m), whereas siRNA knockdown of circCDK1 in KO cells exhibited no such effects (Supplementary Fig. S5 n and o). Taken together, these findings indicated that HNRNPD regulated the biogenesis of circRNAs, and that for a set of genes, including CDK1, the regulation was sufficient to impact the levels of mRNAs and to modulate cellular physiology.

### HNRNPD plays roles in ccRCC cell line

**Figure 6.**
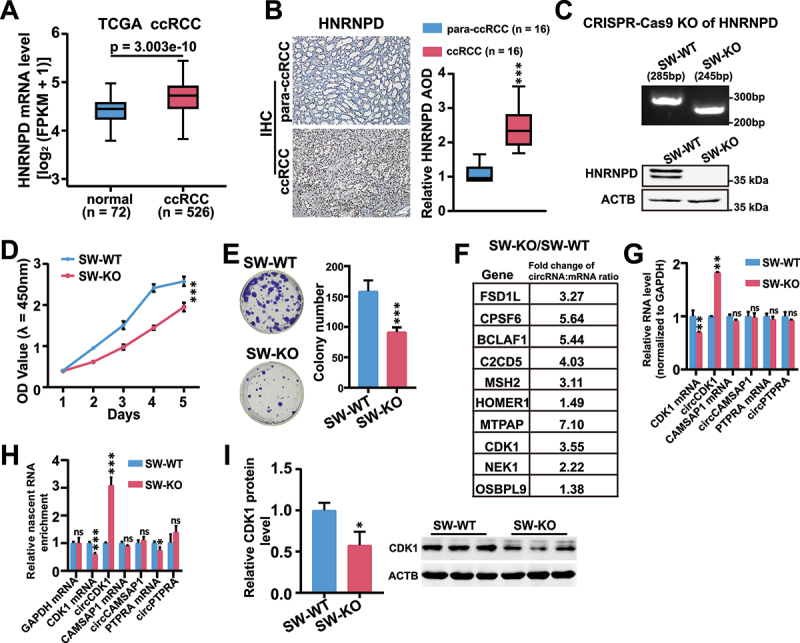
The functions of HNRNPD in ccRCC cells. (A) HNRNPD mRNA level was analyzed in normal people and ccRCC patients from TCGA database. ccRCC, clear cell renal cell carcinoma. *n* = number of people detected. (B) Representative immunohistochemistry (IHC) staining of HNRNPD in para-ccRCC and ccRCC specimens. IHC signal was defined as the average optical density (AOD) quantified by Image Pro Plus software. Scale bar, 100 μm. (C) Validation of HNRNPD KO in SW389 cells. Genomic PCR (upper) and western blot (lower) were used to validate the successful HNRNPD depletion in SW839 cells. (D and E) The cell growth was detected in SW-WT and SW-KO cells by CCK8 assay and colony formation assay. For D, *n* = 5; for E, *n* = 3. (F) Fold change of the ratio of circRNAs and the corresponding mRNAs in SW-KO cells compared to SW-WT cells. Detailed information is provided in the methods section ‘Calculation of the fold change of circRNA: mRNA ratio’. (G and H) RT-qPCR of the expression of circCDK1 and CDK1 mRNA from SW-WT and SW-KO cells at steady and nascent level, respectively. circCAMSAP1 and circPTPRA were selected as negative controls, which were unaffected by the depletion of HNRNPD in RNA-seq data. (I) Western blot of CDK1 in SW-WT and SW-KO cells. Quantification was shown with bar graph. ACTB protein was used as endogenous loading control. *n* = 3. For C – I, SW-WT, SW839 cells; SW-KO, HNRNPD knockout SW839 cells. For A, B, E, and G – I, *p* values from two-tailed student’s *t* test. For D, *p* value from two-way ANOVA test. Data are shown as means ± SD from three independent experiments. *p < 0.05, **p < 0.01, and ***p < 0.001. ns, not significant.

The cellular phenotypes related to the cell cycle and apoptosis in HNRNPD KO cells led us to speculate on the pathological relevance of HNRNPD in the context of circRNA biogenesis. We checked clear cell renal cell carcinoma (ccRCC), as the HEK293T cells used are cells of renal origin. ccRCC accounts for up to 80% of kidney cancers [38], and the majority (75%) of renal cancer-associated deaths [39]. Based on TCGA data, ccRCC tumour specimens have higher HNRNPD mRNA levels than normal specimens (Figure 6(A)). Immunohistochemical (IHC) analysis of 16 pairs of clinical samples that we collected revealed that HNRNPD protein expression was upregulated in ccRCC (Figure 6(B)).

We then used SW839 cells, a ccRCC cell line, for the following experiments. CRISPR-Cas9 was used to generate the HNRNPD KO SW839 (SW-KO) cell line (Figure 6(C)). Cell viability and the colony formation capability were assessed, and both of which were significantly decreased after HNRNPD depletion (Figure 6(D,E)), consistent with the phenotypes of HEK293T KO cells (Figure 1(C,D)). CircRNA-seq analysis of SW-WT and SW-KO cells was then performed to evaluate the steady levels and nascent levels of circRNAs. In accordance with the results from HEK293T cells, both the steady-state levels (Supplementary Fig. S6 a – d) and nascent levels (Supplementary Fig. S6 e – g) of circRNAs were upregulated. Furthermore, we examined the top ten genes exhibiting the highest increase in the circRNA:mRNA ratio in HEK293T cells for their changes in SW839 cells. These 10 genes also exhibited an increased circRNA:mRNA ratio upon HNRNPD KO in SW839 cells (Figure 6(F)). Both the steady-state and nascent levels of circCDK1 were increased, and the CDK1 mRNA and protein levels were conversely decreased upon HNRNPD depletion (Figure 6(G–I)). In addition, the changes in the circRNA:mRNA ratios of the 10 genes were reversed upon HNRNPD overexpression in SW-KO cells, as were the changes in both the steady-state and nascent levels of CDK1 mRNA and circCDK1 (Supplementary Fig. S6 h – j). Taken together, these data indicated that HNRNPD could play regulatory roles similar to those observed in HEK293T cells related to circRNA biogenesis and CDK1 expression also in ccRCC cells.

### Functions of HNRNPD and CDK1 in ccRCC

**Figure 7.**
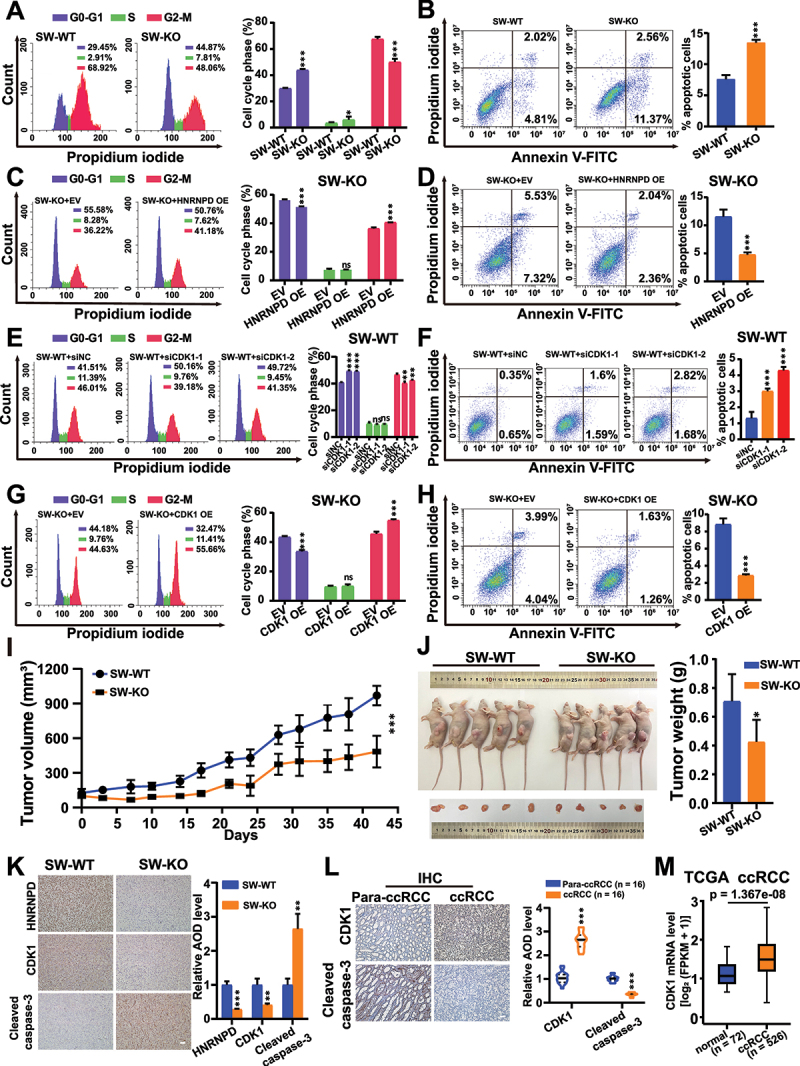
Functions of HNRNPD and CDK1 in ccRCC. (A and B) cell cycle and cell apoptosis were detected by flow cytometry in SW-WT and SW-KO cells. (C and D) Cell cycle and cell apoptosis upon HNRNPD overexpression in SW-KO cells. EV, empty vector. HNRNPD OE, HNRNPD-FLAG overexpression. (E and F) Cell cycle and cell apoptosis in SW-WT cells upon siRNA knockdown of CDK1 mRNA. siNC, siRNA with scrambled sequences. (G and H) Cell cycle and cell apoptosis in SW-KO cells upon CDK1 overexpression. (I) Demonstration of tumor volume at indicated days. The volume of tumors was calculated by the formula: volume = [(length × width^2^)/2]. (J) Photo of tumor and the terminal tumor weight of treated mice. (K) IHC staining of HNRNPD, CDK1 and cleaved caspase-3 in mice tumors. Average optical density (AOD) of IHC signal was calculated by Image Pro Plus software. Scale bar, 100 μm. (L) Representative IHC staining of CDK1 and cleaved caspase-3 in para-ccRCC and ccRCC specimens. IHC signal was defined as the average optical density (AOD) quantified by Image Pro Plus software. Scale bar, 100 μm. (M) CDK1 mRNA level was analyzed in normal people and ccRCC patients from TCGA database. ccRCC, clear cell renal cell carcinoma. *n* = number of people detected. For A – H and K, *n* = 3; for I and J, *n* = 5. For A – K, SW-WT, SW839 cells; SW-KO, HNRNPD knockout SW839 cells. For A – H and J – M, *p* values from two-tailed student’s *t* test. For I, *p* value from two-way ANOVA test. Data are shown as means ± SD from at least three independent experiments. *p < 0.05, **p < 0.01, and ***p < 0.001. ns, not significant.

HNRNPD KO inhibited cell cycle progression and promoted cell apoptosis in SW839 cells (Figure 7(A,B)), while HNRNPD overexpression in SW-KO cells rescued the phenotype (Figure 7(C,D)). CDK1 knockdown in SW-WT cells led to reduced cell cycle progression and increased apoptosis (Figure 7(E,F)), while CDK1 overexpression in SW-KO cells showed the opposite effects (Figure 7(G,H)). CircCDK1 overexpression in SW-WT cells, or circCDK1 knockdown in SW-KO cells, had no significant effect in cell cycle progression or apoptosis (Supplementary Fig. S6 k – n). These results indicated that the cell cycle progression and apoptosis functions of HNRNPD in ccRCC cells were directly linked to its regulation of key genes, such as CDK1, in the context of the mRNA:circRNA ratio.

SW-WT and SW-KO cells were then subcutaneously injected into nude mice (Figure 7(I,J)). The tumours from the KO group showed significantly slower growth, and weighed less (Figure 7(I,J)). IHC staining exhibited a reduced CDK1 level and an increased level of the apoptosis marker cleaved caspase-3 in tumours from the KO group compared to those in the WT group (Figure 7(K)). The CDK1 and cleaved caspase-3 levels were evaluated by IHC in 16 pairs of ccRCC and para-ccRCC specimens, and increased levels of CDK1 and decreased levels of cleaved caspase-3 were observed in the ccRCC samples (Figure 7(L)). Analysis of data from TCGA demonstrated that the levels of CDK1 mRNA were higher in ccRCC tissues than in normal tissues (Figure 7(M)). We also collected 16 paired ccRCC and para-ccRCC specimens to evaluate the levels of CDK1 mRNA and circCDK1 by RT-qPCR analysis. The RT-qPCR analysis revealed that the levels of CDK1 mRNA were higher in the ccRCC specimens than in the para-ccRCC specimens (Supplementary Fig. S6o). Meanwhile, the levels of circCDK1 were lower in the ccRCC specimens than in the para-ccRCC specimens (Supplementary Fig. S6p). In all 16 pairs of clinical samples, the circCDK1:CDK1 mRNA ratio was lower in the ccRCC tumour tissues than in the para-ccRCC tissues (Supplementary Fig. S6q). Taken together, these data demonstrated that HNRNPD exhibited an anti-tumour role in human renal cancer by modulating the ratio of the linear splicing to the backsplicing of key genes, including CDK1.

## Discussion

The precise regulation of ecircRNA biogenesis requires further investigation, and the aftermath of dysregulation in this process needs further characterization. In this study, we revealed the suppressive role of HNRNPD in circRNA biogenesis. Compared to promoting mechanisms, inhibitory regulations in circRNA biogenesis are less understood. The levels of ecircRNAs are primarily increased upon depletion of HNRNPD. HNRNPD preferentially recognizes A-/U-rich motifs, and the flanking introns of the regulated ecircRNAs are longer with more numbers and higher intensity of HNRNPD binding sites. HNRNPD depletion substantially affects the circRNA:mRNA ratio for a small set of genes, and among them, CDK1 is the key gene responsible for the regulatory effects of HNRNPD on cell cycle progression and apoptosis. The regulation of the expression of key genes such as CDK1 by HNRNPD in the context of circRNA biogenesis plays critical roles in cellular physiology, e.g. the cell cycle and apoptosis (Supplementary Fig. S7).

Multiple *cis* elements in pre-mRNAs and *trans* regulatory factors, including some regulatory RBPs, can influence the efficiency of backsplicing [10,11,13,16,17]. Regulatory elements residing in the flanking introns of the circularized exons are closely involved in backsplicing [16,17]. The flanking introns of the HNRNPD-regulated circRNAs harbour more HNRNPD binding sites, and HNRNPD tends to bind to intronic U/A-enriched sites. For a small set of ecircRNAs (46 out of 447), HNRNPD binding sites are also present in the circularized exons (Figure 4J). In canonical splicing, both exonic and intronic binding of specific RBPs, e.g. HNRNPC, can regulate alternative splicing [40,41]. How exonic HNRNPD binding regulates the expression of these circRNAs requires further investigation. A previous study shows that dimerization of certain RBPs, such as QKI and FUS, facilitates circRNA biogenesis by binding to flanking introns and bringing the backsplicing sites close [22,42]. Backsplicing of the *Drosophila* Laccase 2 gene is regulated by combinatorial controls of both intronic repeats and several other HNRNPs and SR proteins [13], which can either positively or negatively influence backsplicing. HNRNPD can dimerize or even oligomerize with its own or with other proteins in interacting with AREs [23]; it is tempting to speculate that the dimerization or oligomerization of HNRNPD may also play roles in ecircRNA biogenesis.

In this study, the perturbation roles of HNRNPD in ecircRNA biogenesis do not alter mRNA profiles at gross levels. Among the five canonical alternative splicing events, IR but not the other four AS events was significantly affected by the reduced level of HNRNPD in the KO cells (Supplementary Fig. S1e). As one example, HNRNPD inhibits the splicing and promotes IR of the E1- and E6- encoding introns on HPV16 early mRNAs [28]. Mechanistically, HNRNPD interacts with components of the splicing machinery, such as U1-70K, U2AF65, and U2AF35, and exerts an inhibitory effect on HPV16 RNA splicing. Both the RRM1 and RRM2 domains of HNRNPD are essential for the interaction of HNRNPD with the intronic region of HPV16 RNA [28]. This study concentrates on the regulatory roles of HNRNPD in backsplicing, and whether HNRNPD utilizes a similar mechanism in regulating the IR of HPV16 RNA in the IR events of mammalian mRNAs requires further investigation.

CircRNAs are generally expressed at low levels compared to their linear counterparts, and backsplicing events are generally inefficient in most cells for most genes [5,6,9]. We find that a set of genes, such as CDK1, exhibits more sensitivity to changes in the HNRNPD level. HNRNPD controls the ratio of linear splicing to backsplicing, and perturbation of HNRNPD expression leads to a significantly changed ratio of ecircRNAs to mRNAs derived from these genes (Figure 5L). Many studies have shown the functions of circRNAs as sponges of miRNAs or are bound to proteins for their functionalities [1,2]. Some circRNAs, especially EIciRNAs, have been shown to regulate host gene transcription [43]. As for circCDK1, we actually do not know its function, but the ratio of circCDK1 to CDK1 mRNA is regulated by HNRNPD, and the altered CDK1 mRNA levels are directly related to cell apoptosis and the cell cycle. Neither overexpression of circCDK1 in WT cells nor knockdown of circCDK1 in HNRNPD KO cells has an effect on cell apoptosis or the cell cycle (Supplementary Fig. S5 j, k, n, o, and Fig. S6 k – n). This finding indicates that circCDK1 does not play a direct role in apoptosis or the cell cycle, but does not exclude the possibility that circCDK1 May exert other biological roles in cells. Also, it is possible that some other circRNAs and mRNAs from key genes sensitive to HNRNPD regulation may take part in the regulatory effects of cellular physiology to some degree.

Thousands of circRNAs in eukaryotes have been identified by high-throughput RNA sequencing and circRNA bioinformatics algorithms, with cell- and tissue-specific expression patterns [44–47]. CircRNAs can also accumulate in specific cell types, especially in non-dividing cells [19,48]. HNRNPD plays similar regulatory roles related to circRNA biogenesis in both HEK293T cells and ccRCC tumour cells. CircRNAs are reported to be generally downregulated in cancers and other diseases, in which the cell proliferation rate is high [1,49]. In this study, HNRNPD depletion leads to increased circCDK1 level and decreased CDK1 mRNA level. Furthermore, ccRCC tumours have higher levels of HNRNPD, lower levels of circCDK1, and higher levels of CDK1 mRNA and protein. We have presented data to show that for the alterations in cell growth and apoptosis observed in either the KO cells or the ccRCC tumours, the key HNRNPD downstream effector is CDK1.

In conclusion, we have identified HNRNPD as a regulatory factor in the biogenesis of ecircRNAs and identified the underlying regulatory mechanism. We have also uncovered a novel functional mechanism by which the regulation of HNRNPD in ecircRNA biogenesis modulates the mRNA levels of key genes to regulate cellular physiology.

## Supplementary Material

Supplemental Material

Supplemental Material

## Data Availability

All data are available in the main article or in the materials. The sequencing reads from this study are deposited into the National Center for Biotechnology Information (NCBI) Gene Expression Omnibus with the accession number GSE212767 (https://www.ncbi.nlm.nih.gov/geo/query/acc.cgi?acc=GSE212767, Secure token: qxwnuwoofbutpit) and GSE240942 (https://www.ncbi.nlm.nih.gov/geo/query/acc.cgi?acc=GSE240942, Secure token: klodukoyfnmjfaj).
