# Supplementary material for: HNRNPD regulates the biogenesis of circRNAs and the ratio of mRNAs to circRNAs for a set of genes
Source: RNA Biol. 2024 Aug 24;21(1):1–15. doi: 10.1080/15476286.2024.2386500 (PMC11346550; doi:10.1080/15476286.2024.2386500)
Supplement: Supplemental Material [file KRNB_A_2386500_SM1273.docx]

**Materials and methods**

**Cell culture**

HEK293T cells were purchased from the American Type Culture Collection (ATCC). SW839 cells were kindly provided by Dr. Bo Peng at the School of Medicine in Tongji University. All cells were cultured in the complete medium (Dulbecco's Modified Eagle's Medium (DMEM, C11995500BT, Gibco) plus 10% FBS (FB25015, CLARK) and 1% penicillin/streptomycin (C0222, Beyotime) at 37 ℃ in a humidified atmosphere containing 5% CO_2_.

**Generation of HNRNPD knockout and HNRNPD^FHBH^ cells by CRISPR-Cas9**

HNRNPD knockout (KO) and HNRNPD^FHBH^ HEK293T cells were generated using the CRISPR-Cas9 genome editing system. For HNRNPD KO HEK293T and SW839 cells, oligonucleotides were annealed and cloned into the linearized (Bsa1) pGL3-U6-sgRNA-PGK-puromycin plasmid. gRNAs targeted the sequences 5’-TGTCGAAGGAACAATATCAG-3’ and 5’- CAACAGTGGGGATCTAGAGG-3’ within the exon 6 (hg19, chr4:83277949-83278048) of HNRNPD (Supplementary Table S1). pST1374-NLS-flag-linker-cas9 plasmid with cloned sgRNAs was transfected into HEK293T and SW839 cells using Lipofectamine 2000 (11668-019, Invitrogen). For HNRNPD^FHBH^ cells, FHBH-tag composed of 3×FLAG, 6×his, 75 AAs that can be biotinylated, and 6×his, was inserted into the C-terminal of endogenous HNRNPD genomic locus using CRISPR-Cas9. gRNAs targeted the sequences 5’-CCATACTAAATTATTCCATTTGC-3’ within the last exon of HNRNPD. The DNA sequence of gRNAs was annealed and cloned into the linearized (Bsa1) pGL3-U6-sgRNA-PGK-puromycin plasmid. The gRNA plasmid, donor plasmid (containing FHBH sequences), and pST1374-NLS-flag-linker-cas9 plasmid were transfected into HEK293T cells using Lipofectamine 2000. After 36 hours, the cells were treated with complete medium (containing puromycin (1 μg/ml), and blasticidin (10 μg/ml)) every two days for one week. Then the cells were cultured without the antibiotic for one week. Finally, the single cell was sorted into 96-well plates using flow cytometer. The deletion in the HNRNPD protein was verified by western blot. For the GFP^FHBH^ cell line, the GFP^FHBH^ expression vector was transfected in 293T cells using Lipofectamine 2000. After 48 hours, the cells were sorted by flow cytometer into 96-well plates. Then the GFP^FHBH^ cells with strong green fluorescence were selected as stable expressed GFP^FHBH^ cells. The cells were cultivated in complete medium at 37 ℃ in a humidified atmosphere containing 5% CO_2_. The cells were passaged by standard methods.

**PCR reactions**

For genomic DNA (gDNA) PCR, gDNA was isolated by phenol/chloroform extraction. For semi-quantitative RT-PCR gels, 25-30 cycles were carried out. RNAs were extracted with TRIzol (15596026, Thermo Fisher Scientific) and treated with DNase I (EN0521, Thermo Fisher Scientific) at 37 ℃ for 30 minutes to digest DNA. RNAs were reverse-transcribed by the GoScript Reverse Transcription System (A5001, Promega) into complementary DNA (cDNA) according to the manufacturer’s protocol. The real-time quantification PCR (RT-qPCR) was performed using GoTaq SYBR Green qPCR Master Mix (A6001, Promega) on a PikoReal 96 real-time PCR system (Thermo). The detailed primer sequences are provided in Supplementary Table S1.

**Western blot**

The protein was separated on SDS-PAGE gels and transferred to PVDF membranes. Then membranes were blocked with 1×TBST (containing 5% skim milk) for 15 minutes. The following steps were according to the western blot manual (GE Healthcare). The membranes were incubated in the primary antibody solution (the primary antibody in 1×TBST buffer containing 5% skim milk) on the shaker overnight at 4 ℃. The primary antibody included the monoclonal mouse antibody against FLAG (The initial concentration was 1 mg/ml, 1:1000 dilution, F1804, Sigma-Aldrich), polyclonal rabbit antibody against HNRNPD (The initial concentration was 1 mg/ml, 1:1000 dilution, PA5-99469, Thermo Fisher Scientific), monoclonal mouse antibody against His (The initial concentration was 1 mg/ml, 1:1000 dilution, M20001S, Abmart), monoclonal mouse antibody against β-actin (The initial concentration was 1 mg/ml, 1:1000 dilution, HC201, TransGen) and polyclonal rabbit antibody against CDK1 (The initial concentration was 550 μg/ml, 1:1000 dilution, 19532-1-AP, Proteintech). The next day, the membranes were rinsed in the 1×TBST buffer 3 times for 5 minutes on the shaker. Then the membranes were incubated in the secondary antibody solution (the secondary antibody in 1×TBST buffer) for 2 hours on the shaker. The secondary antibody included Goat anti-mouse IgG secondary antibody HRP-conjugated (The initial concentration was 1 mg/ml, 1:2000 dilution, L3032, SAB) and Goat anti-rabbit IgG secondary antibody HRP-conjugated (The initial concentration was 1 mg/ml, 1:2000 dilution, L3012, SAB). The membranes were rinsed in the 1×TBST buffer 3 times for 5 minutes on the shaker. Finally, the membranes were added the chemiluminescent substrate and captured chemiluminescent signals using ImageQuant LAS 4000 mini. The intensity of the protein signal was analyzed using Image J software (v1.53a).

**Biotinylated HNRNPD detection**

According to the manufacturer’s manual (K0661, Thermo Fisher Scientific), the biotinylated HNRNPD was detected. Briefly, after the protein was transferred to PVDF membrane, the membrane was washed with Blocking Solution for 30 minutes. Then, the membrane was incubated into the diluted Streptavidin-AP conjugate (Streptavidin-AP conjugate: Blocking solution = 1:5000) for 2 hours at room temperature. After washing three times every 5 minutes, the membrane was added Detection Buffer and performed the enzymatic reaction.

**Immunofluorescence (IF)**

The cells were transferred to 6-well plate, in which the coverslips were put, and cultured overnight until the cells reached 50% confluence. The cells rinsed the ice-cold 1×PBS twice and fixed with cold fixation buffer (methanol: acetic acid = 3:1) for 10 minutes. Aspirating the liquid, and washing with 1×PBS (containing 0.5% Triton X-100) twice on the ice tray for 20 minutes. Aspirating the liquid, the cells were washed three times using 1×PBS (containing 0.1% Tween 20) every 5 minutes. Aspirating the liquid, the cells were blocked in 1×PBS (containing 1% BSA) for 1 hour. Then, the cells were incubated with anti-HNRNPD primary antibody (The initial concentration was 1 mg/ml, 1:200 dilution in 1×PBS containing 1% BSA) at room temperature for 4 hours. The cells were washed with 1×PBS (containing 0.05% Tween 20) three times every 5 minutes, followed by incubation with Alexa Fluor488-labeled secondary antibody (The initial concentration was 2 mg/ml, 1:400 dilution in 1×PBS, ab150077, Abcam) for 2 hours. The cells were washed with 1×PBS (containing 0.05% Tween 20) three times every 5 minutes. Finally, the cells on the slide were stained with DAPI for 15 minutes at room temperature and imaged on a ZEISS LSM880 confocal microscope.

**Library construction, High-throughput sequencing and bioinformatics analysis**

The RNAs from total RNA sample with two replicates were utilized to construct whole transcriptome library by the TruSeq Ribo Profile Library Prep Kit (Illumina, United States), following the manufacturer’s instructions. For circRNA sequencing, linear RNA was digested with 3 U of RNase R (Epicentre, USA) per μg of RNA before library construction. In brief, rRNA was removed by Illumina Ribo-Zero Gold kit, and surplus RNA was purified for end repair and 5′ adaptor ligation. Then, reverse transcription was performed with random primers containing 3′ adaptor. Finally, the cDNAs were purified and amplified with PCR reaction. The products with 300-500 lengths were purified and quantified. These libraries were utilized for 150 nt paired-end sequencing with an Illumina Nova seq 6000 system (Novogene, China). Each library generated ~50 million reads.

To identify circRNAs in HEK293T and SW839 cells, we first trimmed adapters and low-quality reads using cutadapt (v4.2, -m 10) to obtain clean reads. Subsequently, we aligned the reads to the human genome (hg19) using bwa mem (v0.7.17, default parameters). The resulting bam file generated by bwa was then used for circRNA detection with CIRI2 (v2.0.6, -0) [50]. The hg19 GTF file from Genecode (https://www.gencodegenes.org) was provided to CIRI2 for circRNA annotation. Backsplicing junction (BSJ) reads ≥2 were subjected to further analysis and normalized based on sequencing depth. The fold change of circRNAs was calculated using R software, with circRNAs showing abs (log2 (Fold change)) ≥1 considered as differentially expressed.

To analyze linear RNA levels, gene expression levels were calculated from the bam file generated by hisat2 (v2.2.1, default parameters) using featureCounts (v1.6.4) with the Genecode hg19 GTF file.

For data visualization, the boxplots, cumulative fraction curves and MA plots were generated by ggplot2 (v3.3.6) package in R software. The Venn diagrams were generated by VennDiagram (v1.7.3) package in R software.

**Alternative splicing analysis**

For alternative splicing analysis, sequencing of the poly(A) enriched transcripts was performed. In brief, the transcripts of HEK293T cells were enriched by Oligo (dT) magnetic beads. Then, the fragmentation buffer was added to the transcripts to break it into short fragments. The RNA fragments were subject to end repair and 5′ adaptor ligations. Reverse transcription was performed with random primers containing 3′ adaptor sequences. Finally, the cDNAs were purified with AMPure XP beads and amplified with PCR reaction. The PCR product was purified and quantified to obtain the library for 150 nt paired-end sequencing with Illumina Nova seq 6000 system (Novogene, China). Each library was generated a depth of ~50 million reads.

Software rMATs (v4.1.2) was used to calculate the junction reads of AS events including alternative 3′ splice sites (A3SS), alternative 5′ splice site (A5SS), mutually exclusive exon (MXE), intron retention (IR) and skipped exon (SE) in HEK293T cells. The AS events with a false discovery rate (FDR) < 0.05 were selected for further analysis. For data visualization, the boxplots were generated by ggplot2 (v3.3.6) package in R software.

**Fluorescence in situ hybridization (FISH)**

The FISH experiment was modified according to previously published article [51]. TranscriptAid T7 High Yield Transcription Kit (K0441, Thermo Fisher Scientific) was used to generate RNA probes, using the corresponding insertion in the T vector as the template. The 1μg RNA probes were labeled with Alexa Fluor 488 using a ULYSIS Nucleic Acid Labeling Kit (U21652, Invitrogen). The cells were fixed with 4% formaldehyde solution without methanol for 10 minutes and dehydrated sequentially through 70%, 80%, 95% and 100% alcohols for 3 minutes each time. The cells were prehybridization with hybridization buffer (30% formamide, 5×SSC, 9 mM citric acid (pH 6.0), 0.1% Tween 20, 50 μg/mL heparin, 1×Denhardt’s solution and 10% dextran sulfate) at 37 ℃ for 30 minutes Then RNA probe was denatured at 80 ℃ for 10 minutes. Cells were followed by incubation with hybridization buffer and RNA probe at 37 ℃ overnight. Cell slides were washed with washing buffer (5×SSC and 0.1% T Tween 20) at 45 ℃ for 10 minutes twice. Finally, cell slides were stained with DAPI and covered with a coverslip. The slides were observed with confocal microscope (Olympus FV1200). The intensity of the fluorescence signal was analyzed using Image J software (v1.53a).

**Confocal microscopy**

The immunofluorescence (IF) images were acquired using a Zeiss LSM 880 confocal microscope fitted with a 63×1.40 NA oil-immersion objective. Z-stack images were generated at a resolution of 1,024×1,024 using the ZEN Black confocal software (Zeiss, Germany). The resulting images were stored as .czi files. Fluorescence in situ hybridization (FISH) images were captured on an Olympus FV1200 Laser Scanning Confocal Microscope equipped with a GaAsP detector. Z-stack images were acquired using an Olympus IX-71 inverted microscope (Olympus Corporation) with a 60 1.45 NA oil-immersion objective, and the 14-bit digital images were collected using an Andor iXonEM+ DV897K EM CCD camera. The acquired images were saved as .czi files. Subsequently, the .czi files were transformed into .tiff format utilizing the ZEISS ZEN microscope software. Each individual .tiff file underwent processing using Image J software (v1.53a).

**Nascent RNA purification**

To compare the nascent transcript levels in HNRNPD knockout (KO) (HEK293T and SW839) cells and wildtype (HEK293T and SW839) cells, we used the method according to the reference [31]. Briefly, culturing to 80% confluence, the cell transcription was terminated with 0.5 mM 5,6-dichloro-1-β-D-ribofuranosylbenzimidazole (DRB, D1916, Sigma-Aldrich) for 3 hours. Subsequently, the medium was changed to fresh complete medium adding 0.25 mM 5-ethyluridine (EU) for another 1 hour. The cells were washed with ice-cold DPBS three times, and fixed with 90% ethanol for 30 minutes on the ice tray. The cells were washed with cold DPBS three times and merged in the cold DPBS (containing 0.5% Triton X-100) on the ice tray for 15 minutes, and washed with cold DPBS three times. Aspirating the DPBS, the cells were linked up with azide-labeled biotin in the click reaction solution (DPBS, 2.5 mM biotin-azide, 3 mM CuSO4, 6 mM Tris(3-hydroxypropyltriazolymethyl) amine, 10 mM aminoguanidine and 50 mM sodium L-ascorbate) at room temperature for 3 minutes. Aspirating the liquid completely, the cells were washed with DPBS (containing 0.5% Triton X-100 and 2 mM EDTA) 3 times every 3 minutes on the shaker, then washed with DPBS for another two times. Discarding the DPBS, the cells were transferred 1.5 ml tube and suspended with lysis buffer (20 mM Tris-HCl (pH 7.5), 500 mM LiCl, 1 mM EDTA, 0.5% lithium-dodecylsulfate, 5 mM DTT, and 0.1 U/µl RNase inhibitor (N2615, Promega)) on the ice for 20 minutes. The cells were homogenized through a syringe with a 0.4 mm diameter needle five times, and centrifuged at 12,000 g for 10 minutes at 4 ℃. The supernatant was collected into an RNase-free 1.5 ml centrifuge tube. The 5% supernatant was marked as input. The leftover supernatant was immunoprecipitated with streptavidin-conjugated magnetic beads. The magnetic beads were washed with washing buffer and isolated from magnetic beads by RNA elution buffer (10 mM EDTA (pH 8.2) and 95% formamide) at 90 ℃ for 5 minutes, and digested with proteinase K (GE201-01, TransGen) at 56 ℃ for 20 minutes. Finally, the nascent RNAs were extracted with TRIzol.

**Nascent RNA sequencing and data Processing**

The RNAs from nascent RNA samples and corresponding input RNA samples with two replicates were utilized to construct whole transcriptome library by the TruSeq Ribo Profile Library Prep Kit (Illumina, United States). Each library was generated a depth of ~50 million reads. Nascent RNA clean reads of HEK293T were aligned to the human reference genome (hg19) with hisat2 (v2.2.1) using default settings. All duplicates, unmapped reads, reads with more than three mismatches, and non-uniquely mapped reads were removed by samtools (v1.6). MACS2 (v2.2.6) was used to identify peaks of nascent RNA in individual replicates (-q 0.001). The bamCoverage pipeline in deeptools (v3.5.1) was used to generate nascent RNA coverage files (.bw). The files (.bw) from two replicates were merged by bigwigCompare pipeline (--operation mean) for visualization. The common peaks from the replicates were merged using bedtools (v2.30.0). The coverage matrix of each group was calculated with computeMatrix. The heatmap and Metaplot were generated by plotHeatmap and plotProfile pipeline in deeptools, respectively.

Linear RNA and circRNA identification, and differentially expressed circRNAs detection in nascent RNA were in accordance with **Library construction, high-throughput sequencing and bioinformatics analysis** part. The BSJ reads ≥ 1 was subjected to further analysis and normalized by the depth of sequencing.

For data visualization, the boxplots, cumulative fraction curves and MA plots were generated by ggplot2 (v3.3.6) package in R software. The Venn diagrams were generated by VennDiagram (v1.7.3) package in R software.

**RNA stability assay**

The adhered HNRNPD KO HEK293T and the wildtype cells were cultured to 60% confluence in 6-well plate, and treated with 10 µg/ml Actinomycin D. Cells were collected at 0, 6, 12, and 24-hour time points following actinomycin D addition and extracted the RNA with TRIzol reagent.

**FLASH assay**

FLASH was performed as previously described [32]. Briefly, the HNRNPD^FHBH^ cells were washed with 1×PBS and crosslinked by UV irradiation at 200-mJ strength. Then cells were harvested and lysed with 0.5 mL NLB buffer (1×PBS, 0.3 M NaCl, 1% Triton X-100, 0.1% Tween 20) on ice, sonicated, and immunoprecipitated for 10 minutes with 25 μl DynabeadsTM His-Tag Isolation and Pulldown beads (10103D, Thermo Fisher Scientific), which were washed twice with NLB buffer. After the incubation, the beads were washed with NLB buffer and eluted with 250 mM imidazole in NLB buffer for 10 minutes on ice. Elution was collected and incubated with 25 μl M-280 Streptavidin Dynabeads (11206D, Thermo Fisher Scientific) for 2 hours at 4 ℃, after which the beads were washed once with HSB buffer (50 mM Tris-HCl (pH 7.4), 1 M NaCl, 1% IGEPAL CA-630, 0.1% SDS, 1 mM EDTA) and once with NDB buffer (50 mM Tris-HCl (pH 7.4), 100 mM NaCl, 0.1% Tween 20). The beads were then resuspended with 90 μl NDB buffer, to which 10 μl diluted RNaseI (1:200 dilution in NDB buffer, EN0601, Thermo Fisher Scientific) and 2 μl TURBO DNase (AM2238, Thermo Fisher Scientific) were added and treated at 37 ℃ for 3 minutes. The solution was put on ice for 2 minutes, before discarding the supernatant. The beads were washed once with HSB buffer and once with NDB buffer. The beads were resuspended in 20 μl dephosphorylation buffer (10 µl 2×PNK-MES buffer (pH 6.0), 0.1 U RNase inhibitor, 0.1 mM β-mercaptoethanol, 1 U T4 PNK (M0201, NEB)) and incubated at 37 ℃ for 20 minutes. After the dephosphorylation, the beads were washed once with HSB buffer and once with NDB buffer. The beads were then ligated with an s-oligo using T4 RNA ligase I (M0204L, NEB) at 25 ℃ for 1 hour. Excess s-oligo was washed away, and the 3’-phosphate group of the s-oligo was removed with dephosphorylation buffer at 37 ℃ for 20 minutes, after which the beads were washed once with HSB buffer and once with NDB buffer. The complex was resuspended with proteinase K digestion buffer to release crosslinked RNA, and column purification using Oligo Clean&Concentrator (D4060, Zymo Research). The purified RNA was executed reverse transcription with SuperScript III First-Strand Synthesis System (18080-051, Thermo Fisher Scientific), and column purification. The cDNA was then circularized with CircLigase (CL9021K, Lucigen) at 60 ℃ in a hybridization oven overnight. The cyclized cDNA was the template of PCR reaction using NEBNext High-Fidelity Master Mix (M0544, NEB) and FLASH primer (Supplementary Table S1). Then the PCR products were cleaned up with l.5×AM pure beads (A63881, Agencourt). The cDNA library was sequenced using Illumina Nova seq 6000 system platform.

**FLASH data processing**

The FLASH data comprising HNRNPD binding targets was analyzed following the pipeline provided by the previous study [52]. Briefly, we remove the universal adapter from the left sites of reads with bbduk software. Then, we used cutadapt to trim adaptor from both sites of reads to get clean data. We then performed umi_tools to extract 13 nt from 3′ end of sequencing reads to assign as UMI (Unique Molecular Identifiers) tags for every read, accompanied by removing UMI sequences from reads. Reads with UMI tags were aligned to Repbase database to remove suspicious reads that mapped to repetitive DNA. The remaining reads were aligned to the human genome (hg19) with STAR to get a bam format file. We then used *dedup* inside umi_tools to group PCR duplicates and deduplicate reads to yield one read per group. Finally, the clipper software was used to define binding peaks for HNRNPD, and only peaks with supporting sequencing reads of no less than 5 were kept. The HNRNPD binding motifs were identified by Homer (v4.8). We extend 1 nt to the 5' terminal to get specific crosslink site for each peak identified by clipper software. Then we extend 30 nt to both terminals for each crosslink site to get a consistent sequence. Homer was used to identify binding motif for those sequences with default parameters alone with specified motif length 8, 9, and 10 nt (-rna -len 8,9,10). In order to reduce double counting, adjacent peaks within 9 nt were merged by bedtools intersect (v2.30.0). The peak information of location, gene level and gene type were annotated by hg19 GTF file (gencode) with R script. For data visualization, the pie chart was generated by ggplot2 (v3.3.6) package in R software. The circos plots were generated by circlize package (v0.4.13) in R software. The bamCoverage pipeline in deeptools (v3.5.1) was used to generate binding site coverage files (.bw). The files (.bw) for each group were used for IGV (v2.8.10) visualization.

**Identification of the HNRNPD binding sites in the flanking introns of circRNAs**

We firstly found the location of backsplicing exons (circ-Es) from hg19 GTF file (gencode) with R scripts. The upstream intron of 5' circ-E and the downstream intron of 3' circ-E were regarded as flanking intron of circ-Es. Meanwhile, we randomly selected an exon (outside circRNA region) which was not involved in backsplicing (NE), but also from the same set of circRNA generating genes, were analyzed as control. Then, we extracted the flanking intron coordinates of circ-Es and the corresponding NEs from hg19 GTF file (gencode). The binding peak feature of these introns were identified according to FLASH peak data. Those flanking introns of circ-Es and NEs with HNRNPD binding sites were extracted for further analysis. The length and binding site number of these intron was calculated in R software. The HNRNPD binding intensity was defined as the sum of normalized read counts within these flanking introns from FLASH data. To analyze the binding intensity in these flanking introns, the sum of normalized read counts was calculated according to binding site coverage files by deeptools (v3.5.1). For data visualization, the boxplot and barplot was generated by ggplot2 (v3.3.6) package in R software. The .bw files for each group was utilized for IGV (v2.8.10) visualization.

**RNA pull-down and RNA immunoprecipitation**

RNA Pull-down and RNA Immunoprecipitation assays were performed as previously described [53]. The cells were washed with fresh ice-cold 1×PBS three times and crosslinked with 0.12 mJ/cm^2^ UV-C light. Then cells were resuspended in lysis buffer (50 mM Tris-HCl (pH 8.0), 150 mM NaCl, 5 mM EDTA, 1% NP-40, 0.1% SDS, 1 mM DTT, 1×Protease Inhibitor Cocktail, and RNase inhibitor (0.1 U/μl)) for 20 minutes on ice tray. Then the cell lysis was harvested in the 1.5 ml centrifuge tube, sonicated with a Sonics Vibra-Cell (3 seconds on, 6 seconds off, 5 minutes, amplitude 30%) and then centrifuged at 12,000 g for 15 minutes at 4 ℃. The supernatant was collected. The 5% supernatant was marked as input. For RNA pull-down, the leftover supernatant was precleared with M-280 Streptavidin Dynabeads (11206D, Thermo Fisher Scientific) at 4 ℃ for 2 hours. Then the cleared supernatant was collected with magnets and added 200 pmol of biotin-DNA oligonucleotides (Supplementary Table S1) at 4 ℃ for 4 hours. At the same time, M-280 Streptavidin Dynabeads were washed two times with lysis buffer and blocked with yeast total RNA (500 ng/μl) and BSA (1 mg/ml) at 4 ℃ for 4 hours. After the blocked M-280 Streptavidin Dynabeads were washed three times with lysis buffer, the blocked magnetic beads were added to the supernatant mixture at 4 ℃ for 4 hours. For RNA immunoprecipitation, Protein G Dynabeads (10004D, Thermo Fisher Scientific) were initially preincubated with antibodies (2 μg IgG or 2 μg antibody, in the lysis buffer with yeast total RNA (500 ng/μl) and BSA (1 mg/ml)) at room temperature for 2 hours. Then the beads were washed two times with lysis buffer and added leftover supernatant at 4 ℃ for 4 hours. For RNA pull-down and immunoprecipitation, beads were captured with magnets and washed two times with lysis buffer, and three times with lysis buffer supplemented with 500 mM NaCl. The complex was divided into two parts. One part was incubated with deoxyribonuclease (DNase) at 37 ℃ for 20 minutes and 30 μg of proteinase K at 56 ℃ for 20 minutes. Next, the RNA was extracted with TRIzol and performed RT-qPCR. The other part was saved for western bot.

**Mass spectrometry**

The specific silver-strained bands were cut, and digested with trypsin (Promega) in the digestion buffer (100 mM NH4HCO3 (pH 8.5)), and extracted the peptides. Next, the peptides were measured by a Nano LC-ESI-MS/MS system. Protech’s ProtQuest software suite was used to assign the mass values to specific peptide sequences from the UniProt database.

**Gene set enrichment analysis (GSEA)**

The GSEA was performed to annotate the function of nascent genes which can encode circRNAs and be bound by HNRNPD. These genes were pre-ranked by their nascent mRNA expression level. And then the GSEA was implemented in GSEA software (v4.1.0, https://www.gsea-msigdb.org). The levels of nascent mRNAs in WT and KO were normalized to z-score (the number of standard deviations by which the value of a nascent mRNA level is above or below the mean value of what is being measured) in R software. The Venn diagram and heatmap of nascent RNA expression level were generated by ggplot2 (v3.3.6) and pheatmap (v1.0.12) packages in R software.

**Calculation of the fold change of circRNA: mRNA ratio**

The RNA was extracted from the WT, KO, SW-WT, SW-KO, KO + EV, KO + HNRNPD OE, SW-KO + EV, SW-KO + HNRNPD OE cell lines, and 16-paired ccRCC and para-ccRCC specimens. The RNAs were digested with DNase I, and then cDNA was synthesized. The DNA fragments corresponding to circRNA and mRNA were amplified with cDNA and the purified DNA fragments were equally diluted to use to plot standard curves through real-time PCR. The Ct values of circRNA and mRNA in different cells or specimens were also obtained by real-time PCR. Then the amount of circRNA or mRNA in each cell or specimen was determined by utilizing the standard curve. The copy number of circRNA and mRNA in each cell was calculated to obtain the ratio of circRNA: mRNA. The copy number of circRNA was calculated using the following formula: N_circRNA_= X_circ_* 2 * 6.02×10^23^ * the number of cell/ Y_circ._ X_circ_ indicated the amount of circRNA, which was determined by utilizing the standard curve on the basis of standard curves and the Ct value. Y_circ_ indicated the molecular mass of the amplified circRNA fragment. The copy number of circRNA was calculated using the following formula: N_mRNA_= X_m_ * 2 *6.02×10^23^ * the number of cell/ Y_m._ X_m_ indicated the amount of mRNA, which was determined by utilizing the standard curve on the basis of standard curves and the Ct value. Y_m_ indicated the molecular mass of the amplified mRNA fragment. To obtain the ratio of circRNA: mRNA of cell lines or clinic samples, we used the following formula: Ratio_circRNA to mRNA_= N_circRNA_/ N_mRNA_ = (X_circ_ * Y_m_)/ (X_m_ * Y_circ_). The fold change of circRNA: mRNA ratio was calculated by KO Ratio_circRNA to mRNA_: WT Ratio_circRNA to mRNA_, KO+ HNRNPD OE Ratio_circRNA to mRNA_: KO+ EV Ratio_circRNA to mRNA_, SW-KO Ratio_circRNA to mRNA_: SW-WT Ratio_circRNA to mRNA_, SW-KO+ HNRNPD OE Ratio_circRNA to mRNA_: SW-KO+ EV Ratio_circRNA to mRNA_, and ccRCC Ratio_circRNA to mRNA_: para-ccRCC Ratio_circRNA to mRNA_.

**CCK8 assay**

The cells were transferred to the 96-well plate (2000 cells/well). 10 μl CCK8 (40203ES76, YEASEN) was added to each well containing 100 μl complete medium. After 2 hours, the absorbance was detected at 450 nm wavelengths by Multisan GO (Thermo Fisher Scientific).

**Colony formation assay**

For HEK293T cells, 200 HNRNPD KO and wildtype HEK293T cells were cultured in 6-well plates in triplicate for 10 days. For SW839 cells, 600 HNRNPD KO and wildtype SW839 cells were seeded into 6-well plates in triplicate for 15 days. The cells were incubated at 37 ℃ with 5% CO_2_ cell culture incubator. Finally, the cells were stained with 5% crystal violet staining solution (G1063, Solarbio). The colony number was counted by Image J software.

**Cell cycle assay**

The 3×10^5^ cells were inoculated into 6-well plate until cells were attached to the plate. The cells were cultured with complete medium containing 5 mM thymidine for 16 hours. After removing thymidine and washing with fresh medium, the cells were released with complete medium containing 25 μM 2’-Deoxycytidine for 8 hours. Then, the second round of thymidine and 2’-Deoxycytidine were added to cells. After cell synchronization by double thymidine block, the cells were harvested and washed with 1×PBS. The cells were fixed with 75% ethanol overnight. The next day, the cells were washed with 1×PBS twice and stained with Propidium Iodide (PI) (KGA512, KeyGEN BioTECH) for 20 minutes at room temperature in the dark. Finally, the cell cycle was detected by CytoFLEX S Flow Cytometer (Beckman) and was analyzed by CytExpert software.

**Apoptosis assay**

The 3×10^5^ cells were inoculated into 6-well plate until cell confluence reached to 80%. The cells were collected and washed with 1×PBS. Then, the cells were stained with Annexin V and PI according to the manufacturer’s guidelines (KGA108, KeyGEN BioTECH). The apoptotic cells were detected by CytoFLEX S Flow Cytometer (Beckman). The data was analyzed by CytExpert software.

**Xenograft experiment *in vivo***

A group of five mice was fed in one cage and given one week to acclimatize at the Specific-Pathogen-Free (SPF) facility. The mice were 0.1 ml 5×10^6^ HNRNPD KO or wildtype SW839 cells containing 20% Matrigel (BD Biosciences) were injected into 5-week-old male nude mice (SPF Beijing Biotechnology Co. Ltd.) (n = 5 per group), respectively. The male mice were bred in a pathogen-free environment (22 ± 2℃ temperature, 40–60% humidity) with 12 hours light/dark cycle. When the volume of the mouse tumors was no longer decreased, the time was recorded as 0 day. The volume of mouse tumors was measured twice in 7 days. All the mice were sacrificed on the 42nd day, and the tumors were dissected for IHC evaluation. All animal protocols were approved by the Animal Care and Use Committee of the University of Science and Technology of China (USTCACUC23030123009).

**Immunohistochemistry (IHC)**

The 3 μm paraffin sections were in a 70 ℃ oven for 1 hour, then followed by deparaffinized and rehydrated. After antigen retrieval, the sections conducted the removal of endogenous peroxidase in 3% hydrogen peroxide for 10 minutes. The sections were incubated into antibody dilution buffer (ZLI-9029, ZSGB-BIO) at 4 ℃ for 12 hours. Antibodies included polyclonal rabbit antibody against HNRNPD (The initial concentration was 1 mg/ml, 1:100 dilution, PA5-99469, Thermo Fisher Scientific), polyclonal rabbit antibody against CDK1 (The initial concentration was 550 μg/ml, 1:200 dilution, 19532-1-AP, Proteintech), or polyclonal rabbit antibody against Cleaved Caspase-3 (The initial concentration was 52 μg/ml, 1:200 dilution, 9661S, Cell Signaling Technology). The sections were washed with 1×PBS three times, and were stained with secondary antibodies (PV-6000, ZSGB-BIO) at room temperature for 30 minutes. The sections were then stained with 3,3’-diaminobenzidine (DAB), followed by counter-stained with hematoxylin. The excess hematoxylin was rinsed three times. After dehydration and transparency, the sections were sealed with neutral gum. Finally, slides were imaged using TissueFAXS PLUS (TissueGnostics Gmbh). The semi-quantitation of proteins (HNRNPD, CDK1 and cleaved caspase-3) was measured by Image Pro Plus Software 6.0 (Media Cybernetics, CA, United States).

**Clinical samples**

All the ccRCC samples were obtained from the patients who were diagnosed in the Second Affiliated Hospital of Anhui Medical University. This study was reviewed and approved by the Ethics Review Board of the Second Affiliated Hospital of Anhui Medical University (LLSC20190660). Written informed consent was obtained from each patient for this study.

**Statistical Analysis**

In all experiments, we used Student’s *t* test, two-way ANOVA test, Wilcox test, chi-square test and Kolmogorov-Smirnov test to calculate *P* value as indicated in the figure legends. The values reported in the graphs represent averages of three independent experiments, with error bars showing SD. After analysis of variance with F-tests, the statistical significance and *P* values were evaluated with Student’s *t* test.

**Figure legends**

**Figure 1.** Steady-state level of circRNAs increases upon HNRNPD depletion. (A) Scheme for generation of HNRNPD knockout (KO) HEK293T cells by CRISPR-Cas9 system. Forty nucleotides in Exon 6 of HNRNPD were deleted. HNRNPD KO was validated with genomic PCR and western blot (right). β-actin (ACTB) was used as endogenous control. (B) Immunofluorescence (IF) staining of HNRNPD in WT and two KO clone cells. Representative images were shown (DAPI, blue; HNRNPD, green). Scale bar, 20 μm. (C and D) Cell growth was detected by CCK8 and colony formation assay in WT and two KO clone cells. For C, n = 5; For D, n = 3. (E) Venn diagram showed the overlap of steady level of circRNAs from WT and KO cells. (F and G) The boxplots (F) and the cumulative fraction curves (G) of circRNA levels from WT and KO cells. n = 7777. (H) MA plot of the differentially-expressed circRNAs upon HNRNPD KO. circRNAs with at least 2 BSJ reads were used for the analysis. Red plots indicate upregulated circRNAs in HEK293T KO cells. Blue plots indicate downregulated circRNAs in HEK293T KO cells. BSJ, backsplicing junction. (I) Fluorescence *in situ* hybridization (FISH) of circRNAs (circCPSF6 and circASCC3-1) in WT and two KO cells. Representative images were shown (DAPI, blue; circRNA, green). Scale bar, 10 μm. Boxplot showed the relative fluorescence intensity of circRNAs (circCPSF6 and circASCC3-1) in WT and two KO cells. (J) Replicate multivariate analysis of transcript splicing (rMATS) was conducted to analysis the alternative splicing events of the poly(A) enriched transcripts corresponding to differentially-expressed circRNAs in WT and KO cells. SE, skipped exon; A5SS, alternative 5’ splice site; A3SS, alternative 3’ splice site; MXE, mutually exclusive exons; IR, intron retention. The AS events with a false discovery rate (FDR) <0.05 were selected for further analysis. For C, *P* values from the two-way ANOVA test. For D, F and I, *P* values from two-tailed Student’s *t* test. For G, *P* value from the Kolmogorov-Smirnov test. For J, *P* values from the likelihood-ratio test. Data are shown as means ± SD from at least three independent experiments. ****P* < 0.001.

**Figure 2.** Depletion of HNRNPD enhances circRNA biogenesis. (A) Schematic illustration of EU-labeled nascent RNAs purification and measurement. (B) Heatmap represented the normalized level of nascent RNAs in WT and KO cells. The color bar showed the level of nascent RNA. (C) The level of nascent RNAs in WT and KO cells. TSS, Transcription Start Site; TES, Transcription End Site. (D) Venn diagram showed the overlap of nascent level of circRNAs from WT and KO cells. (E and F) The boxplots (E) and the cumulative fraction curves (F) of nascent level of circRNA from WT and KO cells. (G) The comparison of ratios of nascent circRNAs to the overall nascent RNA reads from the same genes. For E and G, *P* values from two-tailed Student’s *t* test. For F, *P* value from the Kolmogorov-Smirnov test.

**Figure 3.** HNRNPD binds to intronic sequences of pre-mRNAs. (A) Scheme for generation of HNRNPD^FHBH^ cell line. The FHBH tag was inserted before the stop codon of HNRNPD genomic DNA by CRISPR-Cas9 knockin. (B) Western blot validation for HNRNPD^FHBH^ protein. HNRNPD has four isoforms: p37, p40, p42, and p45. Detection of four bands or two major bands (one band for p40 & p42, and the other band for p45) was shown. (C) Circos plots exhibited the number and genomic distribution of HNRNPD binding sites. (D) Distribution of HNRNPD binding peaks across different genomic features. The number and the proportion of each part were indicated in the legend. (E) Distribution of HNRNPD binding sites in genomic regions. The number and the proportion of HNRNPD binding sites were indicated. (F) Snapshot of the genomic region with HNRNPD binding. The Refseq genes was shown in the bottom of the snapshots. Group transfected with GFP^FHBH^ was used as the negative control. Kemmerer et al. [27] identified HNRNPD controlled alternative splicing of its exon 9. (G) Comparison of the length of coding genes with or without HNRNPD binding showed by boxplot. n = number of genes detected. (H) Histogram plot of nucleotide frequency within high-confidence HNRNPD-binding peaks (*P* <0.05, reads＞5). (I) Motif analysis of HNRNPD-binding peaks in the intronic and 3’ UTR region of genes. For G, *P* values were from the Wilcoxon test.

**Figure 4.** HNRNPD binding sites have more presence in the flanking introns of circRNAs. (A) Scheme for non-backsplicing exon (NE) and backsplicing exon (circ-E). (B) Percentage of flanking introns (FI) with or without HNRNPD binding for NEs and circ-Es. circ-Es were from the corresponding 7,777 steady circRNAs, while NEs were from the same set of circRNA generating genes. (C) Percentage of FI with HNRNPD binding for NEs and circ-Es. circ-Es were from the corresponding 447 nascent circRNAs, while NEs were from the same set of circRNA generating genes. (D–F) Comparison of the length (D), number of HNRNPD binding sites (E), HNRNPD binding intensity (F) of FI with HNRNPD binding for NEs and circ-Es. Up circ-Es are from circRNAs with increased steady level (Fold change ≥2) in HNRNPD KO HEK293T cells. Non-Up circ-Es are from circRNAs with decreased or unaltered steady level (Fold change <2) in HNRNPD KO HEK293T cells. (G–I) Comparison of the length (G), number of HNRNPD binding sites (H), HNRNPD binding intensity (I) of FI with HNRNPD binding for nascent NEs and circ-Es. Up circ-Es are from circRNAs with increased nascent level (Fold change ≥1.4) in HNRNPD KO HEK293T cells. Non-Up circ-Es are from circRNAs with decreased or unaltered nascent level (Fold change <1.4) in HNRNPD KO HEK293T cells. (J) Cumulative fraction curves were shown the level of WT nascent circRNAs with or without exonic HNRNPD binding. (K and L) Snapshot of the genomic region of CPSF6 (K) and ASCC3-1 (L) with HNRNPD binding. The Refseq gene was shown in the bottom of the snapshots. The circRNA backsplicing junction was connected by grey arc. GFP^FHBH^ group was used as the negative control. For B, C, E and H, *P* values were from chi-square test. For D, F, G and I, *P* values were from two-tailed Student’s *t* test. For J, *P* value was from the Kolmogorov-Smirnov test.

**Figure 5.** HNRNPD modulates the ratio of circRNAs and mRNAs of specific genes. (A) The genes that had nascent mRNA reads detected in WT cells. The percentage of these genes with HNRNPD binding sites from FLASH analysis (left). The percentage of the genes both possessing HNRNPD binding sites and generating circRNAs (right). (B and C) Gene set enrichment analysis (GSEA) demonstration of the enriched pathways for the 264 genes from Figure 5A. (D and E) Flow cytometry was performed to detect cell cycle and cell apoptosis of WT and KO cells. (F and G) Cell cycle and apoptosis of HNRNPD KO cells after HNRNPD overexpression. EV, empty vector. HNRNPD OE, HNRNPD-FLAG overexpression. (H) Venn diagram showed the overlap of the genes relating to cell cycle and apoptosis pathway according to GSEA. (I) Heatmap of the nascent mRNA level of the overlapped genes. Nascent RNA expression level is normalized to Z-score. Fold change of nascent RNA levels of individual gene in HNRNPD KO HEK293T cells compared to WT cells is included in the brackets. (J and K) Bioinformatics analysis of the steady level of circRNAs and corresponding mRNAs from 264 genes in WT and KO cells. The box plot (left) and the cumulative fraction curves (right) demonstrated the steady level of circRNAs (J) and the corresponding mRNAs (K) in WT and KO cells. (L and M) Bioinformatics analysis of the nascent level of circRNAs and corresponding mRNAs from 264 genes in WT and KO cells. The box plot (left) and the cumulative fraction curves (right) demonstrate the nascent level of circRNAs (L) and the corresponding mRNAs (M) in WT and KO cells. (N) The comparison of ratios of normalized circRNA reads to the normalized mRNA reads from these 264 genes. The ratio of RNA-seq was calculated by KO/WT. The increased ratio ＞2; the decreased ratio <1; the unchanged ratio ≥1 and ≤2. Detailed information is provided in the Methods section “Calculation of the fold change of circRNA: mRNA ratio”. For D–G, n=3. For D–G, and J–M (left panels), *P* values from two-tailed Student’s *t* test. For J–M (right panels), *P* values from the Kolmogorov-Smirnov test. Data are shown as means ± SD from three independent experiments. **P* < 0.05, ***P* < 0.01, and ****P* < 0.001.

**Figure 6.** The functions of HNRNPD in ccRCC cells. (A) HNRNPD mRNA level was analyzed in normal people and ccRCC patients from TCGA database. ccRCC, clear cell renal cell carcinoma. n = number of people detected. (B) Representative immunohistochemistry (IHC) staining of HNRNPD in para-ccRCC and ccRCC specimens. IHC signal was defined as the average optical density (AOD) quantified by Image Pro Plus software. Scale bar, 100 μm. (C) Validation of HNRNPD KO in SW389 cells. Genomic PCR (upper) and western blot (lower) were used to validate the successful HNRNPD depletion in SW839 cells. (D and E) The cell growth was detected in SW-WT and SW-KO cells by CCK8 assay and colony formation assay. For D, n = 5; For E, n = 3. (F) Fold change of the ratio of circRNAs and the corresponding mRNAs in SW-KO cells compared to SW-WT cells. Detailed information is provided in the Methods section “Calculation of the fold change of circRNA: mRNA ratio”. (G and H) RT-qPCR of the expression of circCDK1 and CDK1 mRNA from SW-WT and SW-KO cells at steady and nascent level, respectively. circCAMSAP1 and circPTPRA were selected as negative controls, which were unaffected by the depletion of HNRNPD in RNA-seq data. (I) Western blot of CDK1 in SW-WT and SW-KO cells. Quantification was shown with bar graph. ACTB protein was used as endogenous loading control. n = 3. For C–I, SW-WT, SW839 cells; SW-KO, HNRNPD knockout SW839 cells. For A, B, E, and G–I, *P* values from two-tailed Student’s *t* test. For D, *P* value from two-way ANOVA test. Data are shown as means ± SD from three independent experiments. **P* < 0.05, ***P* < 0.01, and ****P* < 0.001. ns, not significant.

**Figure 7.** Functions of HNRNPD and CDK1 in ccRCC. (A and B) Cell cycle and cell apoptosis were detected by flow cytometry in SW-WT and SW-KO cells. (C and D) Cell cycle and cell apoptosis upon HNRNPD overexpression in SW-KO cells. EV, empty vector. HNRNPD OE, HNRNPD-FLAG overexpression. (E and F) Cell cycle and cell apoptosis in SW-WT cells upon siRNA knockdown of CDK1 mRNA. siNC, siRNA with scrambled sequences. (G and H) Cell cycle and cell apoptosis in SW-KO cells upon CDK1 overexpression. (I) Demonstration of tumor volume at indicated days. The volume of tumors was calculated by the formula: volume = [(length × width^2^)/2]. (J) Photo of tumor and the terminal tumor weight of treated mice. (K) IHC staining of HNRNPD, CDK1 and cleaved caspase-3 in mice tumors. Average optical density (AOD) of IHC signal was calculated by Image Pro Plus software. Scale bar, 100 μm. (L) Representative IHC staining of CDK1 and cleaved caspase-3 in para-ccRCC and ccRCC specimens. IHC signal was defined as the average optical density (AOD) quantified by Image Pro Plus software. Scale bar, 100 μm. (M) CDK1 mRNA level was analyzed in normal people and ccRCC patients from TCGA database. ccRCC, clear cell renal cell carcinoma. n = number of people detected. For A–H and K, n = 3; For I and J, n = 5. For A–K, SW-WT, SW839 cells; SW-KO, HNRNPD knockout SW839 cells. For A–H and J–M, *P* values from two-tailed Student’s *t* test. For I, *P* value from two-way ANOVA test. Data are shown as means ± SD from at least three independent experiments. **P* < 0.05, ***P* < 0.01, and ****P* < 0.001. ns, not significant.

**References:**

[1] Gao Y, Zhang J, Zhao F. Circular RNA identification based on multiple seed matching. Brief Bioinform. 2018;19(5):803–810. doi: 10.1093/bib/bbx014

[2] Chen L, Wang Y, Lin J, et al. Exportin 4 depletion leads to nuclear accumulation of a subset of circular RNAs. Nat Commun. 2022;13(1):5769. doi: 10.1038/s41467-022-33356-z

[3] Van Nostrand EL, Pratt GA, Shishkin AA, et al. Robust transcriptome-wide discovery of RNA-binding protein binding sites with enhanced CLIP (eCLIP). Nat Methods. 2016;13(6):508–514. doi: 10.1038/nmeth.3810

[4] Gao L, Chang S, Xia W, et al. Circular RNAs from BOULE play conserved roles in protection against stress-induced fertility decline. Sci Adv. 2020;6(46):eabb7426. doi: 10.1126/sciadv.abb7426
