# Supplementary material for: HNRNPD regulates the biogenesis of circRNAs and the ratio of mRNAs to circRNAs for a set of genes
Source: RNA Biol. 2024 Aug 24;21(1):1–15. doi: 10.1080/15476286.2024.2386500 (PMC11346550; doi:10.1080/15476286.2024.2386500)
Supplement: Supplemental Material [file KRNB_A_2386500_SM0666.zip › Supplementary data_highligted.docx]

**Supplemental material**

**Supplementary Figure S1**. Validation of circRNAs and alternative splicing analysis. (A) RT-qPCR validation of the upregulated circRNAs and corresponding mRNAs identified in the circRNA sequencing. circDnmt1 and circPCNX1 were used as the negative control. (B) RT-qPCR analysis of circRNAs and their corresponding mRNAs after RNase R treatment. (C) Demonstration of the backsplicing sites of circRNAs by sanger sequencing using divergent primers. The arrows show the backsplicing sites of circRNAs. The genomic coordinates of circRNAs were also shown. (D) RT-qPCR analysis of circRNAs and their corresponding mRNAs in two HEK293T KO cells upon overexpression of HNRNPD. EV, empty vector. HNRNPD OE, HNRNPD-FLAG overexpression. (E) Replicate multivariate analysis of transcript splicing (rMATS) was conducted to analyze the differential alternative splicing events identified from sequencing the poly(A)-enriched transcripts in WT and KO cells. SE, skipped exon; A5SS, alternative 5’ splice site; A3SS, alternative 3’ splice site; MXE, mutually exclusive exons; IR, intron retention. The AS events with a false discovery rate (FDR) < 0.05 were selected for further analysis. For A, B and D, n = 3. For A, B and D, *P* values from two-tailed Student’s *t* test. For E, *P* values from the likelihood-ratio test. Data are shown as means ± SD from three independent experiments. **P* < 0.05, ***P* < 0.01, and ****P* < 0.001. ns, not significant.

**Supplementary Figure S2**. Validation of circRNA biogenesis and the degradation. (A) RT-qPCR validation of nascent circRNAs and their corresponding nascent mRNAs. GAPDH mRNA was a negative control. (B) RT-qPCR validation of nascent levels of circRNAs and their corresponding mRNAs in two HEK293T KO cells after overexpression of HNRNPD. EV, empty control. HNRNPD OE, HNRNPD-FLAG overexpression. (C–I) RT-qPCR validation of circRNAs after actinomycin D treatment at indicated time in WT and two KO cells. For A–I, n = 3. For A and B, *P* values are from two-tailed Student’s *t* test. For C–I *P* values from the two-way ANOVA test. Data are shown as means ± SD from three independent experiments. **P* < 0.05, ***P* < 0.01, and ****P* < 0.001. ns, not significant.

**Supplementary Figure S3**. Demonstrations of HNRNPD binding and the impact of HNRNPD-FHBH on circRNAs and the cell growth. (A) Schematic procedure of FLASH assay. (B–F) Snapshot of the genomic region of ASCC3-2 (B), DDHD1 (C), MAPK1 (D), ADGRL2 (E) and BRIP1 (F) with HNRNPD binding. The Refseq gene was shown in the bottom of the snapshots. The circRNA backsplicing junction was connected by grey arc. GFP^FHBH^ group was used as the negative control. (G) RT-qPCR examination of steady levels of circRNAs and their corresponding mRNAs in two HEK293T KO cells after overexpression of HNRNPD-FHBH. EV, empty control. n = 3. (H) The cell growth was detected after overexpression of HNRNPD-FHBH in HEK293T KO cells by CCK8 assay. n = 5. For G, P values from two-tailed Student’s t test. For H, P values from two-way ANOVA test. Data are shown as means ± SD from at least three independent experiments. **P* < 0.05, ***P* < 0.01, and ****P* < 0.001. ns, not significant.

**Supplementary Figure S4**. HNRNPD interacted with circCPSF6 and circASCC3-1. (A) Schematic procedure of the circRNA pulldown for interacting proteins. (B and C) Pulldown of circCPSF6 and circASCC3-1 in HEK293T cells. Pulldown efficiency of circCPSF6 and circASCC3-1 were shown in the bar graph. Silver staining of the pulled-down proteins and western blot of HNRNPD were shown. red bracket denotes the HNRNPD band. Scr, negative control oligo with scrambled sequences; Oligo, biotin-labeled antisense oligos against the junction of circCPSF6 and cirASCC3-1, respectively. (D and E) The identified unique peptide of HNRNPD from mass spectrometry of circCPSF6 and circASCC3-1 pulldown. (F) HNRNPD RNA IP (RIP) of the interacting circRNAs. The relative RIP enrichment of circRNAs and western blot of HNRNPD IP are shown. For B, C and F, Data are shown as means ± SD from three independent experiments. *P* values from two-tailed Student’s *t* test, ***P* < 0.01, and ****P* < 0.001. ns, not significant.

**Supplementary Figure S5**. The functions of HNRNPD and CDK1 in HEK293T cells. (A) Venn diagram of the identified genes in WT and KO cells. (B) Fold change of the ratio of circRNA and the corresponding mRNA in HEK293T WT and KO cells upon HNRNPD overexpression. Detailed information is provided in the Methods section “Calculation of the fold change of circRNA: mRNA ratio”. EV, empty vector. HNRNPD OE, HNRNPD-FLAG overexpression. (C and D) RT-qPCR validation of the expression of circCDK1 and CDK1 mRNA from WT and KO cells at steady and nascent level, respectively. circCAMSAP1 and circPTPRA were selected as negative controls, which were unaffected by the depletion of HNRNPD in RNA-seq data. (E) Westen blot of CDK1 protein in HEK293T WT and KO cells. Quantification was shown with bar graph. ACTB protein was used as endogenous loading control. (F and G) RT-qPCR analysis of the steady-state and nascent levels of circCDK1 and CDK1 mRNA in KO cells after overexpression of HNRNPD. (H and I) Cell cycle and apoptosis in 293T cells upon CDK1 knockdown. Two siRNAs were used to target CDK1 mRNA. siNC, siRNA with scrambled sequences. (J and K) Cell cycle and apoptosis of 293T cells upon circCDK1 overexpression. EV, empty vector. (L and M) Cell cycle and apoptosis of KO cells upon CDK1 overexpression. EV, empty vector. (N and O) Cell cycle and apoptosis in KO cells upon circCDK1 knockdown. Two siRNAs were used to target circCDK1. siNC, siRNA with scrambled sequences. For B–O, n = 3. For C–O, *P* values from two-tailed Student’s *t* test. Data shown represent the means ± SD from three independent experiments. **P* < 0.05, and ****P* < 0.001. ns, not significant.

**Supplementary Figure S6.** HNRNPD modulates the ratio of circRNAs and mRNAs in ccRCC. (A) Venn diagram showing the overlap of circRNAs detected in SW-WT and SW-KO cells. (B and C) The boxplots (B) and the cumulative fraction curves (C) of circRNA levels in SW-WT and SW-KO cells. (D) MA plot illustrating the expression changes of circRNAs in SW839 cells upon HNRNPD KO. CircRNAs with at least 2 BSJ reads were used for the analysis. Red plots indicate the upregulated circRNAs upon HNRNPD knockout. Blue plots indicate the downregulated circRNAs upon HNRNPD knockout. BSJ, backsplicing junction. (E) Venn diagram showing the overlap of nascent circRNAs in SW-WT and SW-KO cells. (F and G) The boxplots (F) and the cumulative fraction curves (G) of the nascent circRNA levels in SW-WT and SW-KO cells. (H) Fold change of the ratio of circRNA and the corresponding mRNA in SW-KO cells upon HNRNPD overexpression. EV, empty vector. HNRNPD OE, HNRNPD-FLAG overexpression. (I and J) RT-qPCR analysis of the steady-state (I) and nascent (J) levels of circCDK1 and CDK1 mRNA in SW-KO cells after HNRNPD overexpression. (K and L) Cell cycle and apoptosis of SW-WT cells after circCDK1 overexpression. (M and N) Cell cycle and cell apoptosis in SW-KO cells upon circCDK1 siRNA knockdown. (O and P) RT-qPCR analysis of the expression of CDK1 mRNA and circCDK1 in para-ccRCC and ccRCC specimens. (Q) The fold change of the circCDK1: CDK1 mRNA ratio in 16 paired ccRCC versus para-ccRCC specimens. N., number. For H and Q, detailed information is provided in the Methods section “Calculation of the fold change of circRNA: mRNA ratio”. For A–N, SW-WT cells, SW839 cells; SW-KO cells, HNRNPD knockout SW839 cells; For I–N, Data shown represent the means ± SD from three independent experiments. For B, F and I–P, *P* values from two-tailed Student’s *t* test. For C and G, *P* values from the Kolmogorov-Smirnov test. ***P* < 0.01, and ****P* < 0.001. ns, not significant.

**Supplementary Figure S7.** A working model of HNRNPD in modulating the biogenesis of ecircRNA and the physiological relevance. HNRNPD inhibits backsplicing, which leads to lower levels of ecircRNA biogenesis and higher levels of linear splicing. For a set of genes including CDK1, this regulatory effect of HNRNPD is potent to substantially affect the mRNA levels. Through the regulation of key target genes e.g. CDK1, HNRNPD exerts its physiological functions in cells and ccRCC.
