# Supplementary material for: HNRNPD regulates the biogenesis of circRNAs and the ratio of mRNAs to circRNAs for a set of genes
Source: RNA Biol. 2024 Aug 24;21(1):1–15. doi: 10.1080/15476286.2024.2386500 (PMC11346550; doi:10.1080/15476286.2024.2386500)
Supplement: Supplemental Material [file KRNB_A_2386500_SM0666.zip › Table S2_csh.docx]

**Supplementary Table S2. Oligos used in the study**

| KO-sgHNRNPD-F1 | ACCGCTGATATTGTTCCTTCGACA |
| --- | --- |
| KO-sgHNRNPD-R1 | AAACTGTCGAAGGAACAATATCAG |
| KO-sgHNRNPD-F2 | ACCGCAACAGTGGGGATCTAGAGG |
| KO-sgHNRNPD-R2 | AAACCCTCTAGATCCCCACTGTTG |
| HNRNPD-KO-test-F | AAACATGTTGGCTATTCTGG |
| HNRNPD-KO-test-R | CCTCCATAACCACCGTAACC |
| KI-sgHNRNPD-F | ACCGGCAAATGGAATAATTTAGTA |
| KI-sgHNRNPD-R | AAACTACTAAATTATTCCATTTGC |
| HNRNPD-Donor-F1 | AGGCCCCAGTCAAAACTGGA |
| HNRNPD-Donor-R1 | CCGTCATGGTCTTTGTAGTCGTATGGTTTGTAGCTATTTTG |
|  |  |
| HNRNPD-Donor-F2 | CAAAATAGCTACAAACCATACGACTACAAAGACCATGACGG |
|  |  |
| HNRNPD-Donor-R2 | AAGTTGCAAATGGAATAATTTAGTGATGATGATGATGATG |
|  |  |
| HNRNPD-Donor-F3 | CATCATCATCATCATCACTAAATTATTCCATTTGCAACTT |
|  |  |
| HNRNPD-Donor-R3 | TGATACAAAAAATTTAGTTTG |
| HNRNPD-KI-test-F | CACTTGGGAATTAAGCTACCC |
| HNRNPD-KI-test-R | CACAAGACTTGCTCTACAATAC |
| circCPSF6-FISH-F | GCAGAGAACGAGAGAGGCAC |
| circCPSF6-FISH-R | TAATACGACTCACTATAGGGGGCATCTCCATTATTTGCAG |
|  |  |
| circASCC3-1-FISH-F | AGCCACTAGGCATCATTGTG |
| circASCC3-1-FISH-R | TAATACGACTCACTATAGGGTGTTGTAGGCAGTCTCAAAC |
|  |  |
| Q-GAPDH-F | CTTCATTGACCTCAACTACATGG |
| Q-GAPDH-R | CTCGCTCCTGGAAGATGGTGAT |
| Q-circCPSF6-F | GCAGAGAACGAGAGAGGCAC |
| Q-circCPSF6-R | GGCATCTCCATTATTTGCAG |
| Q-circASCC3-1-F | AGCCACTAGGCATCATTGTG |
| Q-circASCC3-1-R | TGTTGTAGGCAGTCTCAAAC |
| Q-circDDHD1-F | CCCATATTGTATTTGTTGTG |
| Q-circDDHD1-R | CTCTTCTAGAGGCTGCCAAG |
| Q-circMARK1-F | CACAGACTGTGAAAATCTTC |
| Q-circMARK1-R | TACTCGACTTGGTAGGCTGG |
| Q-circ ADGRL2-F | TCTTCTGGTTGCAGAATGCG |
| Q-circ ADGRL2-R | GAATCTAGTTATAAATGCCC |
| Q-circASCC3-2-F | AGTAAGGATTCCCTACAGCG |
| Q-circASCC3-2-R | TCAGACTGAATAGTGACTTG |
| Q-circBRIP1-F | TTCCAAGATGAAGTGGGAGC |
| Q-circBRIP1-R | CGATGACTCTTGACTGTTTC |
| Q-circ PITPNB-F | CTACAAAGCTGATGAAGACC |
| Q-circ PITPNB-R | TACGCATTCCAGGCTTTCTC |
| Q-circRPS15A-F | GCCTAAGAAACAGTCACCCCT |
| Q-circRPS15A-R | CCACACTTGTTTAGCCTGCC |
| Q-circPCNX-F | CACTTTTGGCCCTGTTGAT |
| Q-circPCNX-R | TCTTCTGTTGGGCCGTAAGT |
| Q-circDnmt1-F | TGTAATCCTGGGGCTAGGTGAAGG |
| Q-circDnmt1-R | CCAAATCGGATGAGTCCATCAAGG |
| Q-circCDK1-F | TTCTTTCCATGGATCTGAAG |
| Q-circCDK1-R | CTTCCATAGTTAGTCAATGG |
| Q-circCAMSAP1-F | CGTTCAGTGCCTCGAAAGAAC |
| Q-circCAMSAP1-R | ACAGGCGGCTTAATGTGCT |
| Q-circPTPRA-F | AACCAGTTCACGGATGCCAG |
| Q-circPTPRA-R | GCATTGTTGGCACTGACACAT |
| Q-circFSD1L-F | CCTGGTTTCATGAAGTGGTACA |
| Q-circFSD1L-R | CAGCACTGTGTATGATTCTCCA |
| Q-circBCLAF1-F | GGAAACTGCAAAGACTGGGA |
| Q-circBCLAF1-R | TGCAGGATCAAGAAGTCAAGTG |
| Q-circC2CD5-F | CGTGGAAGGCTGTTTGGAAC |
| Q-circC2CD5-R | ACGTGCACTAACTACACCCC |
| Q-circMSH2-F | TGACTCCTCTTACTGATCTTCGT |
| Q-circMSH2-R | CACAACACTCTGCAGATTCTTTG |
| Q-circHOMER1-F | CAACGGGACAGATGATGAAAGA |
| Q-circHOMER1-R | TTGGAAGACATGAGCTCGAG |
| Q-circMTPAP-F | TATTCGACAGGGAAGGGAGC |
| Q-circMTPAP-R | AGAGTGTTCAGCTGATCGTCT |
| Q-circNEK1-F | AGAAGAGTTCCTGCAGCGAA |
| Q-circNEK1-R | TCCACTGCCCAGAAAAGGAG |
| Q-circOSBPL9-F | ATCAAAGTGGCTCATCCCCA |
| Q-circOSBPL9-R | TCATTCCATCTGCGTGCTTC |
| Q-CPSF6-mRNA-F | TGGCGGACGGCGTGGACCAC |
| Q-CPSF6-mRNA-R | GATGGAGATATGACATCGTC |
| Q-ASCC3-mRNA-F | ACTGGAGAAGAGTAAAATGC |
| Q-ASCC3-mRNA-R | CCAACAGAGTCCTTCAAGTG |
| Q-DDHD1-mRNA-F | AAATACTGGAAGTCAAGACC |
| Q-DDHD1-mRNA-R | AAGTATTGTACCAGTGGATC |
| Q-MARK1-mRNA-F | CAGTTGGATCAAAAAGCGAG |
| Q-MARK1-mRNA-R | CAATGCTACGTATCGATCTG |
| Q-ADGRL2-mRNA-F | CTCTGCATGATTTCCACTGG |
| Q-ADGRL2-mRNA-R | TATGTTTAGCCAGTTCATTG |
| Q-BRIP1-mRNA-F | AATATACAATTGGTGGGGTG |
| Q-BRIP1-mRNA-R | AAAGCAGAACAAAGTAAGGC |
| Q-PITPNB-mRNA-F | CGTGTGGTTTTGCCATGTTC |
| Q-PITPNB-mRNA-R | CCATCCTTCTCATAAGGTTC |
| Q-RPS15A-mRNA-F | TGAGTTTTGGGGTCCAGCAG |
| Q-RPS15A-mRNA-R | GCCAAGTGCGCTTTACACAA |
| Q-CDK1-mRNA-F | CACTTGGCTTCAAAGCTGGC |
| Q-CDK1-mRNA-R | ATAGTTAGTCAATGGGTATG |
| Q-CAMSAP1-mRNA-F | AGTGAACGAACCCCACAAGA |
| Q-CAMSAP1-mRNA-R | TAGTAGCAGTAAAGCGCCCTG |
| Q-PTPRA-mRNA-F | GGAATGTGAGAGCTACACCGT |
| Q-PTPRA-mRNA-R | CGGATCTGCCGGCTCTTATT |
| Q-FSD1L-mRNA-F | TTTGATGGGGGTCAACTTTCATT |
| Q-FSD1L-mRNA-R | AGTGTTCTCACAGCACTTGGA |
| Q-BCLAF1-mRNA-F | ACCAAGGGGATGGGATTGTT |
| Q-BCLAF1-mRNA-R | TGGGTGCAAGTTCTGCTCTG |
| Q-C2CD5-mRNA-F | GTGAGAGCTCATGTTGCTGC |
| Q-C2CD5-mRNA-R | GGCAGTTGGTAGTAGGTTGCT |
| Q-MSH2-mRNA-F | AGAAAGCCCTGGAACTTGAGG |
| Q-MSH2-mRNA-R | TGTTTCACCTTGGACAGGAACT |
| Q-HOMER1-mRNA-F | CACTGAAACTGAAGGAAGAGGAAA |
| Q-HOMER1-mRNA-R | CCCTCCAGGTCTTTGTTCCG |
| Q-MTPAP-mRNA-F | AACCTTCACAGAACACAGAAACA |
| Q-MTPAP-mRNA-R | TCAGGTTTGTTTTGCTCCCT |
| Q-NEK1-mRNA-F | GGCAGATGGAGCCTACCAAG |
| Q-NEK1-mRNA-R | TGCTGTATTTCCCACTGAAGC |
| Q-OSBPL9-mRNA-F | CAGAACGAGTATGAATCCCGC |
| Q-OSBPL9-mRNA-R | CTTCAAGCCTGTGCTTTGCT |
| s-oligo | [Phosphate]NNCACUUGNYYNNagatcggaagagcgtcgt[18 atom spacer]acgtgtgctcttccgatct[Phosphate] |
| 5'biotin-Scr | TTCTCCGAACGTGTCACGTTCGAACGTGTC |
| 5'biotin-circCPSF6 | ACCATATTCAGCTTCCTTCTAACGATGACG |
| 5'biotin-circASCC3-1 | AGCCAGCTGTCCGATCTGAGTTCGTAAAAT |
| FHBH-sequence | ATGGACTACAAAGACCATGACGGTGATTATAAAGATCATGACATCGATTACAAGGATGACGATGACAAGCATCATCATCATCATCACgccggtaaggccggagagggcgagattcccgctccgctggccggcaccgtctccaagatcctcgtgaaggagggtgacacggtcaaggctggtcagaccgtgctcgttctcgaggccatgaagatggagaccgagatcaacgctcccaccgacggcaaggtcgagaaggtccttgtcaaggagcgtgacgccgtgcagggcggtcagggtctcatcaagatcggcCATCATCATCATCATCAC |
| FLASH-F | AATGATACGGCGACCACCGAGATCTACACTCTTTCCCTACACGACGCTCTTCCGATCT |
| FLASH-R | CAAGCAGAAGACGGCATACGAGATTGACCACCGTGACTGGAGTTCAGACGTGTGCTCTTCCGATCT |
| siNC | UUCUCCGAACGUGUCACGUTT  ACGUGACACGUUCGGAGAATT |
| siCDK1-1 | UGUUAAUCUAUGAUCCAGCC  GGCUGGAUCAUAGAUUAACA |
| siCDK1-2 | ACAAUCAGAUUAAGAAGAUG  CAUCUUCUUAAUCUGAUUGU |
| sicircCDK1-1 | ACUUGUUAAGGAUCUACCAU  AUGGUAGAUCCUUAACAAGU |
| sicircCDK1-2 | CACUUGUUAAGGAUCUACCA  UGGUAGAUCCUUAACAAGUG |
